# *Vibrio cholerae* motility exerts drag force to impede attack by the bacterial predator *Bdellovibrio bacteriovorus*

**DOI:** 10.1038/s41467-018-07245-3

**Published:** 2018-11-12

**Authors:** Miles C. Duncan, John C. Forbes, Y Nguyen, Lauren M. Shull, Rebecca K. Gillette, David W. Lazinski, Afsar Ali, Robert M. Q. Shanks, Daniel E. Kadouri, Andrew Camilli

**Affiliations:** 1 Department of Molecular Biology and Microbiology, Tufts University School of Medicine, Boston, MA 02111 USA; 2 Harvard-Smithsonian Center for Astrophysics, Cambridge, MA 02138 USA; 3 Emerging Pathogens Institute, University of Florida, Gainesville, FL 32608 USA; 4 Department of Environmental & Global Health, School of Public Health and Health Profession, University of Florida, Gainesville, FL 32610 USA; 5 Department of Ophthalmology, Campbell Laboratory of Ophthalmic Microbiology, University of Pittsburgh, Pittsburgh, PA 15213 USA; 6 Department of Oral Biology, Rutgers School of Dental Medicine, Newark, NJ 07101 USA; 7 Present Address: Department of Microbiology, University of Texas Southwestern Medical Center, Dallas, TX 75390 USA

## Abstract

The bacterial predator *Bdellovibrio bacteriovorus* is evolved to attack and kill other bacteria, including the human intestinal pathogen *Vibrio cholerae*. Although *B*. *bacteriovorus* exhibit a broad prey range, little is known about the genetic determinants of prey resistance and sensitivity. Here we perform a genetic screen on *V. cholerae* and identify five pathways contributing to predation susceptibility. We find that the essential virulence regulators ToxR/S increase susceptibility to predation, as mutants of these genes are more resistant to predation. We observe by flow cytometry that lipopolysaccharide is a critical defense, as mutants lacking O-antigen are rapidly attacked by predatory *B. bacteriovorus*. Using polymer solutions to alter media viscosity, we find that when *B. bacteriovorus* attacks motile *V. cholerae*, increased drag forces slow its ability to prey. These results provide insights into key prey resistance mechanisms, and may be useful in the application of *B. bacteriovorus* in treating infections.

## Abstract (web summary)

Prey bacteria have evolved different strategies to counteract predation but the genetic basis remains unclear. Here, Duncan et al. identify key genes involved in *Vibrio cholerae* sensitivity to *Bdellovibrio bacteriovorus* predation, providing new insights into prey resistance mechanisms.

## Introduction

B*dellovibrio bacteriovorus* is a predatory bacterium capable of preying on a wide array of Gram-negative bacteria including human pathogens and is often described as a living antibiotic^1^. These small bacteria first hunt and then attach to their prey via mechanisms thought to involve motility and type IV pili (T4P) respectively^2,3^. Within 10–20 min of attachment, *B. bacteriovorus* invades the prey periplasm where it kills the host and remodels host peptidoglycan to create a spherical bdelloplast^4^. Inside this protected niche, it degrades the host’s cytosolic proteins and nucleic acids, using these products to fuel its own replication before it lyses its prey and releases several daughter cells^5^. This lifecycle is usually completed within 2–3 h, underscoring *B. bacteriovorus*’ potential as an anti-infective therapy. Owing to the alarming rise of antibiotic-resistant bacteria and dearth of new treatments entering the clinic, *B. bacteriovorus* is being evaluated for therapeutic purposes and has recently been shown to attenuate *Klebsiella pneumoniae* and *Shigella flexneri* in vivo^6,7^.

Another susceptible prey species is *Vibrio cholerae*^8,9^, the causative agent of the severe diarrheal disease cholera. The global cholera burden is substantial, with 3–5 million annual cases leading to over 100,000 deaths each year^10^. The disease is endemic in parts of the Americas, Africa, and South Asia, and recent epidemics have occurred following natural disasters in Haiti. Disrupted water infrastructure can increase disease burden as the pathogen lives in marine and brackish waters in many temperate parts of the world^11^. Considering that *B. bacteriovorus* is ubiquitous in water and soil, it likely preys on *V. cholerae* in its natural environment^12^.

While *B. bacteriovorus*’ basic lifecycle is well established, the genetic mechanisms of predation for prey and predator remain poorly understood^13^. One study, focusing on the predation of *Erwinia caratova* and *Pseudomonas syringae*, found that resistance was plastic rather than genetic and could be quickly reversed^14^. However, a recent study demonstrated that under certain conditions *Chromobacterium piscinae* produces cyanide to protect itself from *B. bacteriovorus* predation^15^. To further explore potential genetic differences in susceptibility, we created saturated transposon libraries in *V. cholerae* and *Escherichia coli*, subjected them to predation by *B. bacteriovorus*, and used transposon-insertion sequencing (Tn-seq) of the surviving prey to identify susceptibility and resistance genes. We identified very few such genes in *E. coli*, but many in *V. cholerae*, suggesting that prey species may vary widely in their evolved strategies to resist predation by *B. bacteriovorus*.

Here we report five pathways important for increased or reduced sensitivity of *V. cholerae* to *B. bacteriovorus* predation. Two pathways sensitize *V. cholerae* to predation, as these mutants are more resistant to *B. bacteriovorus* killing. A key resistance mechanism is lipopolysaccharide (LPS), as mutants lacking O-antigen are attached to and killed more rapidly by *B. bacteriovorus*. Additionally, we find that drag exerted by the motion of *V. cholerae* slows the predator’s ability to kill its prey.

## Results

### Tn-seq for prey genes involved in predation

To identify prey pathways playing a role in predation, we created mTn*10* transposon libraries in *V. cholerae* and *E. coli* (Supplementary Data 1) and subjected them to predation by *B. bacteriovorus*. The *V. cholerae* library contained 50,000 unique insertions while the *E. coli* library contained 80,000 unique insertions, such that both libraries approached saturation. For each of three biological replicates, we prepared two parallel culture tubes with 10^9^colony-forming units (CFU)/ml prey, infecting one with *B. bacteriovorus* at a multiplicity of infection (MOI) of 0.1 for *V. cholerae* and MOI 0.5 for *E. coli*. For *V. cholerae*, we observed 0.6% survival after 14 h of predation, while *E. coli* was more sensitive to *B. bacteriovorus* and showed 0.4% survival after just 3 h. To avoid sequencing contaminating DNA from dead prey, we outgrew all of the prey that survived the infection (≈5 × 10^6^ CFU) in 200 ml LB broth overnight, while in parallel outgrowing 5 × 10^6^ CFU of the uninfected prey in 200 ml LB broth as well.

We next isolated genomic DNA (gDNA) from each sample and processed the DNA for massively parallel sequencing of the mTn*10* junctions using Illumina sequencing. A new method termed Nextera Tn-seq was devised for sample processing, which uses Illumina Nextera Tagmentation to place a defined sequence adjacent to mTn*10* insertions to allow for subsequent PCR amplification of the transposon junctions (see Methods for details). Each PCR product was comprised of a unique 8 base pair index adjacent to the mTn*10* inverted repeat, an unknown gDNA sequence of variable length flanking the transposon, and Illumina-specific sequence necessary for sequencing (Supplementary Data 2). We combined the DNA samples to allow multiplex sequencing and sequenced the DNA using the Illumina Hiseq 2500 to determine the frequency of each transposon insertion in the infected and uninfected populations. We calculated the fitness contribution of each gene using the bioinformatics software as previously described^16^, where fitness represents the net survival of that gene disruption mutant relative to the bulk population (Supplementary Data 3 and 4).

**Fig. 1:**
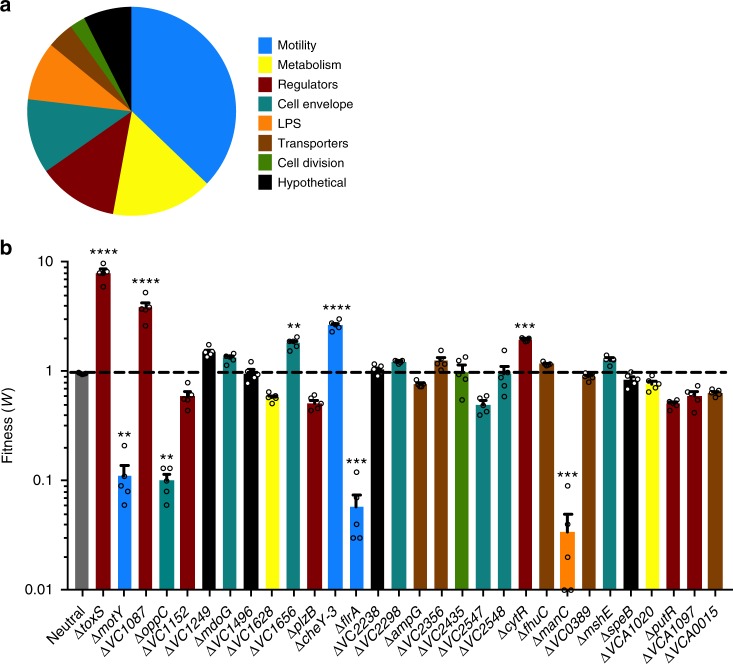
*V. cholerae* mutants with altered sensitivity to *B. bacteriovorus* predation. **a** We generated a complex *V. cholerae* transposon mutant library and subjected it to transposon-insertion sequencing (Tn-seq) before and after predation by *B. bacteriovorus*. Mutants with decreased fitness (*w* < 0.4) are shown and categorized according to gene ontology terms. **b** Promising transposon mutants identified in **a** were re-created by gene deletion and replacement with an FRT scar. This FRT scar served as a pseudo-transposon for a mini Tn-seq of a much smaller mutant library: 32 mutants compared to 50,000 in the initial Tn-seq. The color scheme of **a** matches **b**. The average fitness values and standard errors of the mean (SEM) for five biological replicates are shown. Error bars represent standard error of the mean. Significance was determined by comparing the fitness of each mutant to the average fitness values for two neutral genes. **P < 0.002; ***P < 0.0007; ****P < 0.0001 (ANOVA and Dunnett’s multiple comparisons test)

Using gene ontology terms, we categorized the 151 insertions that reduced *V. cholerae* fitness <0.4 (Fig. 1a), indicating that these transposon mutants had a survival defect during *B. bacteriovorus* predation. Over one third of the reduced fitness mutants had insertions in flagellar motility genes, in part a reflection of the many genes required to construct and regulate a flagellum. These genes included the master transcriptional regulator FlrA and the flagellar motor protein MotY. Other prominent gene categories included metabolism, general regulatory genes, cell envelope genes, and LPS genes. The majority of genes required for O-antigen biosynthesis and transport were hits. Seventy-nine *V*. *cholerae* gene insertions demonstrated improved survival during predation with fitness values >2.5, including the virulence regulators ToxR and ToxS^17^ (Supplementary Data 3).

The *E. coli* reduced-fitness hits were less promising as there were only 14 gene insertions producing fitness values <0.4 (Supplementary Data 4). Furthermore, the smallest fitness value was 0.26, whereas the *V. cholerae* screen demonstrated 71 hits with fitness values <0.1 (Supplementary Data 3). The *E. coli* Tn-seq screen resulted in 69 gene insertions with fitness values >2.5, but none of these hits validated in later experiments.

### Validation of *V. cholerae* gene hits

To validate the *V. cholerae* hits from the Tn-seq screen, we created a small library of representative marked gene deletion mutants and performed a mini Tn-seq screen on the pooled mutants. We used the FRT/FLP recombinase system to create 32 *V. cholerae* mutants, each with an identical 81 bp FRT scar in place of the gene of interest^18,19^. As controls, this library included several mutants with initial fitness values between our cutoffs of 0.4 and 2.5 (VC2298, AmpG, PutR, VCA1097), as well as two neutral mutants with fitness values close to 1.0 in the initial Tn-seq. We performed the selection for the screen as described above, with five biological replicates for each pair of infected and uninfected prey *V. cholerae*. The FRT scar served as a pseudo-transposon for junctional sequencing^19^, and thus the sample preparation, sequencing, and bioinformatics analysis were repeated as described above for the ten new samples.

Following the mini Tn-seq screen, 9 of the 26 mutants tested for validation showed significantly increased or decreased fitness values when compared to the neutral genes (Fig. 1b). Both non-motile mutants ∆*flrA* and ∆*motY* showed reduced fitness, while of the two only the ∆*motY* mutant makes a flagellum (Supplementary Figure 1). This suggests flagellum presence is irrelevant to predation and it is motility of wild-type (WT) prey that alters resistance to *B. bacteriovorus*. Other genes required for resistance to predation included OppC, a transmembrane component of the ABC transporter permease system for small peptides^20^, and ManC, a type II phosphomannose isomerase required for O-antigen biosynthesis^21^ (Supplementary Figure 2). Genes that in WT prey increase sensitivity to predation (these mutants were more resistant) included the virulence regulator ToxS and the response regulator VC1087^17,22^. The majority of these mutants showed similar motility to WT *V. cholerae* in soft agar chemotaxis assays (Supplementary Figure 3). For follow-up experiments, we decided to focus on the 5 prey pathways that demonstrated fitness values <0.4 or >2.5 in the mini Tn-seq.

**Fig. 2:**
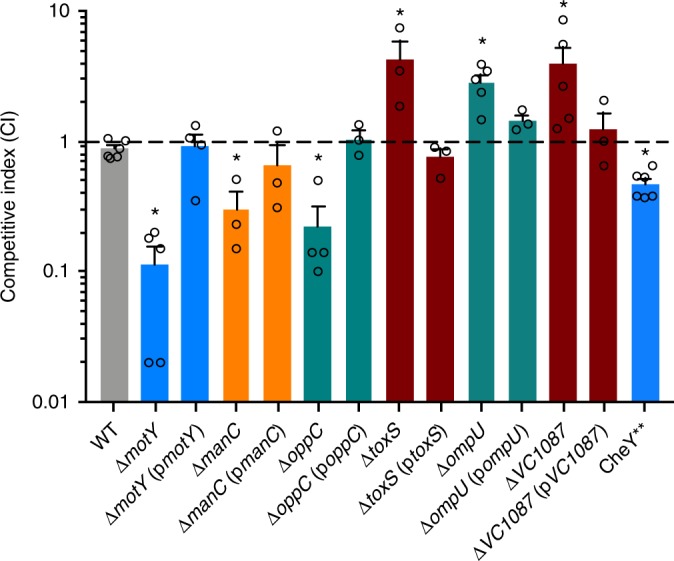
*V. cholerae* complementation restores WT sensitivity to predation. In 1:1 competitions with WT, *V. cholerae* mutants showed similar sensitivity to *B. bacteriovorus* predation as in the previous Tn-seq experiments. Complemented strains showed WT levels of sensitivity to predation. CheY**, a tumble-biased strain, showed intermediate sensitivity to predation compared to WT and ∆*motY* strains. The average competitive indices and standard error of the mean for three to six biological replicates are shown. *P < 0.05 (ANOVA with Dunnett’s multiple comparisons test)

To confirm these mutants did not harbor off target mutations, we complemented each representative strain by cloning their respective open reading frames into the plasmid pMMB67EH and transferring these plasmids to each mutant parent strain. Additionally, we created a mutant with a tumble-biased version of the chemotaxis protein CheY (CheY**)^23^ to determine whether prey swimming speed or style affects susceptibility to predation. We confirmed the CheY** mutant both had a severe tumble bias compared to the smooth swimming phenotype of the WT by microscopy and defective chemotaxis in the soft agar assay (Supplementary Figure 3). We also created a mutant of the outer membrane porin OmpU and a complemented strain. ToxR/S positively regulates *ompU* expression^17^, thus we aimed to test whether reduced OmpU expression could explain predation resistance by the ∆*toxS* strain. Next we competed these strains 1:1 with an isogenic ∆*lacZ V. cholerae* strain during *B. bacteriovorus* predation and plated the survivors on 5-bromo-4-chloro-3-indolyl-b-D-galactopyranoside (X-gal) plates to enumerate blue and white CFUs. Complementation restored WT sensitivity to predation (Fig. 2). In addition, the tumble-biased CheY** mutant showed an intermediate phenotype, as it was more sensitive to predation than WT *V. cholerae* but less sensitive than the non-motile ∆*motY* mutant. The ∆*ompU* strain was also more resistant to predation, similar to the ∆*toxS* mutant.

### *B. bacteriovorus* rapidly attach to O-antigen minus prey

We hypothesized differences in mutant *V. cholerae* susceptibility to *B. bacteriovorus* might be due to attachment rates. We used flow cytometry on fluorescent bacteria to gauge direct predator–prey interaction. We first generated a constitutive green fluorescent protein (GFP) construct and transformed it into the *V. cholerae* strains. For *B. bacteriovorus*, we used a previously created strain harboring a tdTomato-expressing plasmid^24^. Following 30 min of infection at an MOI of 1, we fixed the samples before diluting into phosphate-buffered saline (PBS) and analyzing by flow cytometry. We reasoned that any events deemed double positive (green and red) would indicate attachment or interaction between *B. bacteriovorus* and *V. cholerae*. The presence of such double-positive complexes was confirmed by sorting the two populations and using standard fluorescence microscopy (Supplementary Figure 4). To account for false-positive events, we fixed one set of samples immediately upon mixing, which was well before *B. bacteriovorus* could attach to *V. cholerae*.

**Fig. 3:**
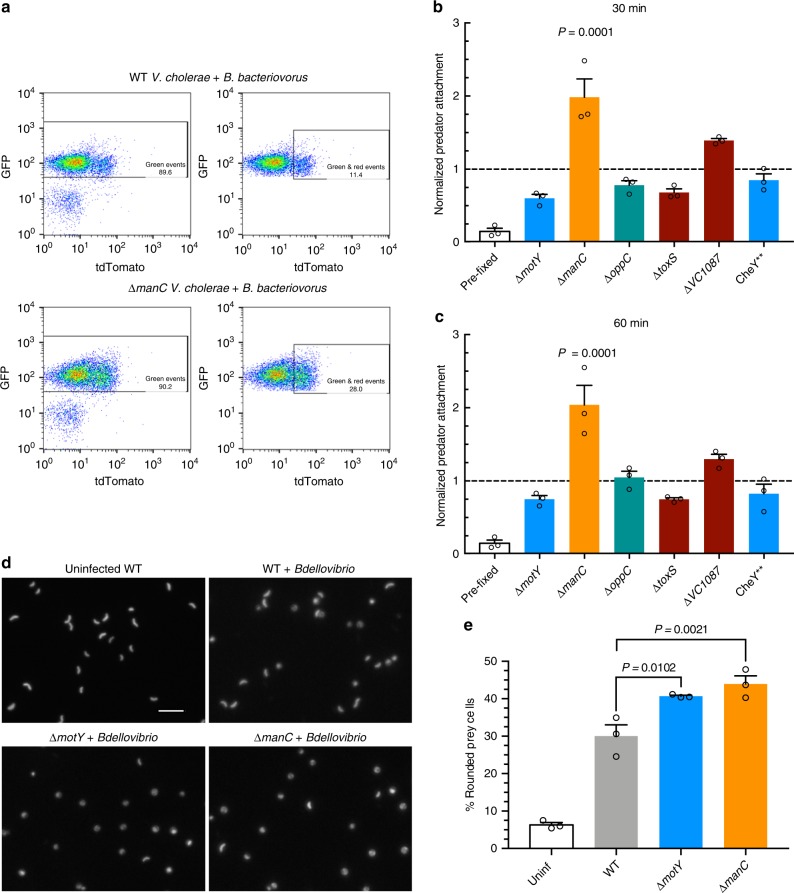
*V. cholerae* rounding and attachment by *B. bacteriovorus*. **a** Gating strategy for analyzing *B. bacteriovorus* attachment to *V. cholerae* by flow cytometry. The left plots show all events, while right plots show green-only and double-positive events for the same experiment. **b**, **c** Predator attachment to prey by flow cytometry at MOI 1 at 30 min (**b**) or 60 min (**c**) post infection. As a control, fluorescent *B. bacteriovorus* and *V*. cholerae were fixed immediately upon mixing to account for false positive interactions. All attachment to mutant prey was normalized to WT prey attachment for three biological replicates. **d** Fluorescence images of GFP-expressing *V. cholerae* 1 h following infection with *B. bacteriovorus* at MOI 1. The scale bar indicates 10 µm. **e** The percentage of rounded *V. cholerae* cells was calculated by analyzing images by Matlab for roundness (eccentricity) of three biological replicates. Error bars represent standard error of the mean. Statistics were analyzed by ANOVA with Dunnett’s multiple comparison’s test

We collected 10,000 events for each sample and applied a gating strategy to limit false positives (Fig. 3a, Supplementary Figure 5). Surprisingly, the majority of *V. cholerae* mutants did not show a significant change in *B. bacteriovorus* attachment compared to WT prey (Fig. 3a–c). The exception was the ∆*manC* mutant, which does not produce O-antigen and showed a two-fold higher attachment rate. This suggests that normal *V. cholerae* O-antigen might interfere with predator attachment. We observed the same results at 30 min (Fig. 3b) or 60 min (Fig. 3c) postinfection.

### The ∆*motY* and ∆*manC* strains are rounded more than WT prey

Since the majority of *V. cholerae* mutants did not show alterations in predator attachment, we hypothesized that the predation cycle of *B. bacteriovorus* is altered in the mutant prey. If true, we would predict prey rounding, the first step following *B. bacteriovorus* attachment and entry, might be altered for the mutants. To test this, we infected GFP-expressing WT, ∆*manC*, and ∆*motY V. cholerae* with *B. bacteriovorus* at an MOI of 1 for 1 h. We then fixed the cells and imaged the *V. cholerae* by fluorescence microscopy (Fig. 3d). Next we used a custom Matlab script to analyze each bacterium’s circularity by calculating its eccentricity. Eccentricity scores can range from zero to one, with perfect circles scored as zero. For our Matlab script, we used a cutoff of 0.4, where any bacteria below that number were considered bdelloplasts and any bacteria >0.4 were considered unrounded vibroid-shaped cells.

After analyzing a minimum of 3300 cells across three biological replicates, our script identified 6% of uninfected cells as rounded and 30% of infected WT *V. cholerae* cells as rounded (Fig. 3e, Supplementary Figure 6). These ratios matched our visual observations of the images, validating our eccentricity cutoff of 0.4. Both ∆*motY* and ∆*manC V. cholerae* were significantly more rounded than WT, with >40% of these mutant cells scoring <0.4 for eccentricity. This ∆*manC* data is consistent with the higher predator attachment shown in Fig. 3b, c. However, the greater rounding of the non-motile ∆*motY* mutant suggests that its increased susceptibility to predation manifests post-attachment and that swimming motility by WT *V. cholerae* slows progression of the *B. bacteriovorus* lifecycle.

### Drag forces slow *B. bacteriovorus* predation of *V. cholerae*

The attachment phase of *B. bacteriovorus* is reported to last about 10–20 min, before the predator is able to invade its host’s periplasm^13^. However, we suspected this process may be extended or interrupted for highly motile prey-like *V. cholerae*, whose motion should exert a strong drag force on the attached predator. Prey bdelloplast formation occurs shortly after predator invasion, so this might explain why non-motile mutant prey are rounded and killed more than motile prey (Fig. 3d, e). To calculate the difference in force on *B. bacteriovorus* attached to the two types of prey, we employed a variation of Stokes’ law (Eq. 2), which is used for calculating drag at low Reynolds numbers, with a modification to account for the non-spherical shape of the bacteria^25^:

$$F_{{\mathrm{drag}}} = 3{\mathrm{\pi }}\eta v(d_{\mathrm{n}})(1/3 + (2/3)d_{\mathrm{s}}/d_{\mathrm{n}})$$

Here *η* represents the medium’s dynamic viscosity, *v* is velocity, and *d*_n_ and *d*_s_ represent the equivalent spherical diameters of, respectively, the object’s cross-sectional area along the direction of motion and the object’s surface area. Using this equation, we find that, when attached to *V. cholerae* traveling at 80 µm/s (Supplementary Figure 7), *B. bacteriovorus* experiences 0.32 piconewtons (pN) of drag force. Comparatively, non-motile *V. cholerae* travel at approximately 1 µm/s due to Brownian motion^26^, meaning any attached *B. bacteriovorus* would only experience 0.004 pN of drag force. We also observed *V. cholerae* dragging *B. bacteriovorus*, at an average speed of 29.4 µm/s, under a ×90 objective by phase-contrast microscopy (Supplementary Movies 1–4). Using this *V. cholerae* speed with an attached predator, we calculate that the *B. bacteriovorus* would face 0.12 pN of drag force.

To further explore the role of drag forces on *B. bacteriovorus* predation, we used ficoll and methylcellulose, polymers that increase medium viscosity to a greater extent than they slow bacterial swimming speed^27–29^. We used ficoll at 10%, which increases the medium viscosity from 0.77 centipoise (cP) to 4.03 cP at 30 °C. This 5.23-fold viscosity increase was accompanied by a 2.5-fold decrease in *V. cholerae* velocity, calculated by tracking individual bacteria over time by time-lapse microscopy (Supplementary Figure 7). Since the drag force is proportional to the product of velocity and viscosity, we expect that *B. bacteriovorus* attached to *V. cholerae* will experience roughly a factor of 2.1 greater drag in the high-viscosity condition, all else being equal. We also used the polymer methylcellulose at 1%, which increased viscosity to 3.06 cP.

**Fig. 4:**
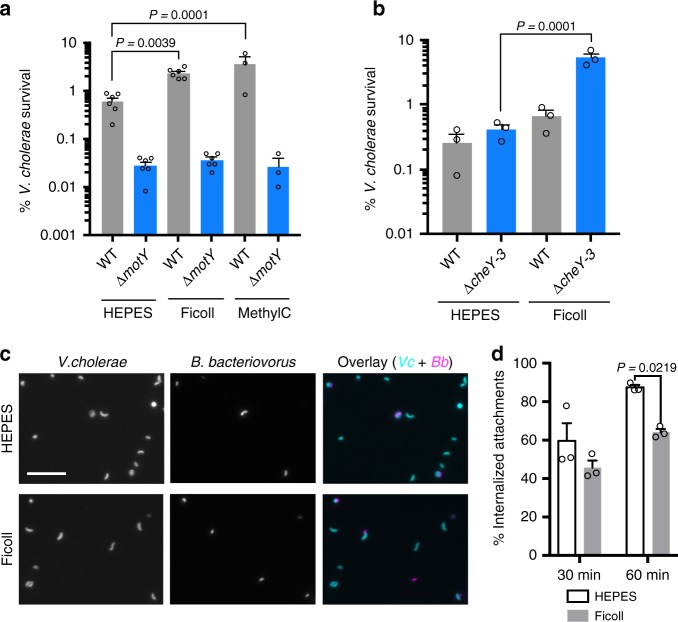
Increasing viscosity reduces killing and invasion of motile *V. cholerae*. **a**, **b** The survival percentage of motile and non-motile *V. cholerae* strains in HEPES medium alone or supplemented with ficoll or methylcellulose to increase viscosity. Infections were carried out for 14 h with an MOI of 0.1. Survival percentage and SEM is shown for three to six biological replicates. **c** Fluorescence microscopy of *B. bacteriovorus* (magenta) and *V. cholerae* (cyan), following 1 h of infection at MOI 0.1 in HEPES or Ficoll-supplemented medium. Scale bar indicates 10 µm. **d** Images from **c** were scored for externally attached or internalized *B. bacteriovorus* at 30 and 60 min. The percentage of internalized attachments and SEM is shown for three biological replicates. Error bars represent standard error of the mean. Statistics were analyzed by ANOVA with Dunnett’s multiple comparisons test

We then performed 1:1 competitions between a ∆*lacZ* mutant and isogenic WT or ∆*motY V. cholerae* in plain HEPES medium or medium supplemented with ficoll or methylcellulose. Consistent with our hypothesis, the WT strain was four- and six-fold more resistant to *B. bacteriovorus* killing at these higher viscosities, respectively, while the non-motile strain ∆*motY* showed the same survival rate regardless of medium viscosity (*P* = 0.999, Fig. 4a). The increased viscosity led to a small, but not significant, decrease in predator–prey attachment for both WT and ∆*motY* prey (Supplementary Figure 8). We also tested ∆*cheY3*, a strain locked in the smooth-swimming state that does not pause to tumble, in the higher viscosity predation conditions (Fig. 4b). As expected, in ficoll the ∆*cheY3* strain showed an even greater competitive advantage over WT prey. We hypothesize these mutants exert more consistent drag force on attached *B. bacteriovorus* as they do not stop to tumble like WT *V. cholerae*. The increased viscosity did not alter the competitive index for the ∆*lacZ V. cholerae* used in the competition experiments (Supplementary Figure 9).

Finally, we used fluorescent *V. cholerae* and *B. bacteriovorus* to observe whether higher viscosity alters predator invasion of prey (Fig. 4c). We infected WT *V. cholerae* with *B. bacteriovorus* at MOI 0.1 for 30 min or 1 h and then fixed the cells for fluorescence microscopy. For each of the three biological replicates, we scored 200 *B. bacteriovorus*, double blind, for invasion or external attachment to *V. cholerae* in HEPES or 10% ficoll medium (Fig. 4d). While at 30 min, we only noticed a trend, at 1 h we observed significantly less predator attachments that had progressed to prey invasion. These data are consistent with the finding that non-motile prey are more rounded (Fig. 3e) than WT and suggests that high drag forces slow *B. bacteriovorus* prey invasion and killing.

## Discussion

First identified by Hans Stolp in 1962, *B. bacteriovorus* has long been studied for its unique predatory lifestyle and therapeutic potential against drug-resistant pathogens^30^. Several years after its discovery, Varon and Shilo reported cell wall mutant prey with varied *B. bacteriovorus* attachment rates^31^. However, in the intervening years little research has focused on prey genes involved in the predation process, nor has a specific receptor for *B. bacteriovorus* been identified in any prey species. To address these questions, we used Tn-seq to identify prey genes with positive or negative roles in susceptibility to predation. Although we found no evidence for a specific receptor, we did find five prey pathways that influence susceptibility to *B. bacteriovorus* predation. Using flow cytometry, we show that *V. cholerae* mutants lacking O-antigen are attached to more easily by *B. bacteriovorus*. We also find that both O-antigen mutant and non-motile mutant *V. cholerae* are rounded into spherical bdelloplasts more than WT prey at 1 h postinfection. Additionally, we propose that motile prey exert increased drag forces on attached *B. bacteriovorus*, slowing predator entry and killing compared to non-motile prey.

In our attachment assay, only the O-antigen-defective ∆*manC* mutant showed significantly increased interaction between *V. cholerae* and *B. bacteriovorus*. It is interesting to note that, while LPS might serve as a defense against *B. bacteriovorus*, it is often a receptor for bacteriophages that prey on *V. cholerae*^21,32^. These opposing selective pressures likely play out in the aquatic environment and possibly even in the human host. While none of the other *V. cholerae* mutants showed significantly altered attachment, the non-motile ∆*motY* strain did trend toward less *B. bacteriovorus* interactions than WT. This is consistent with kinetic theory whereby motile prey should collide with motile *B. bacteriovorus* more frequently^33^.

Despite standard predator attachment, the ∆*motY* mutants are rounded into bdelloplasts more than motile WT prey. We propose that this is due to the drag force exerted by motile *V. cholerae* on attached *B. bacteriovorus*, as increasing this drag force with ficoll or methylcellulose improved survival rates for motile *V. cholerae* and reduced predator invasion. Currently, it is not known what *B. bacteriovorus* proteins or mechanisms mediate attachment^34,35^ or the force this attachment can withstand. *V. cholerae* motility may exert sufficient drag force to shear the predators off of their prey, and though we did not observe this process in action, we did observe *B. bacteriovorus* attached to still-moving *V. cholerae*. An alternative hypothesis is that the angle of attachment is crucial for *B. bacteriovorus* invasion, and drag force prevents the predator from crossing the prey cell’s outer membrane. The ratio of runs to tumbles in motility appears to play a role in prey defense as well. For instance, the smooth-swimming-biased ∆*cheY3* strain demonstrated nearly two-fold increased resistance to predation, whereas the tumble-biased CheY** strain showed decreased resistance to predation. These data suggest that *B. bacteriovorus* may opportunely invade prey when they slow down and there is a concomitant drop in shear force.

It is unclear why genes of the oligopeptide permease Opp were identified in our screen, but mutants of this transporter were significantly more sensitive to predation. This permease is involved in peptidoglycan recycling, so it is possible that *B. bacteriovorus* is better able to remodel or cross the host peptidoglycan layer upon entry into the periplasm^20,36^. The critical virulence regulators ToxR/S were also involved, as these mutants were more resistant to predation. It is noteworthy that environmental *V. cholerae* may face evolutionary pressure against ToxR/S from *B. bacteriovorus*, but losing these regulators would see them lose the ability to cause disease in the human host^37^. Similarly, mutants of the response regulator VC1087 were more resistant to predation, and this gene has been linked to *V. cholerae* virulence^22^. ToxR/S regulates over 20 genes in *V. cholerae*, yet all of these genes, except for *ompU*, had neutral fitness values in our Tn-seq screen (Supplementary Data 3). Our data suggest that OmpU may promote predation, as ∆*ompU* and ∆*toxS* strains, which produce less OmpU, were both resistant to predation. This result contrasts with previous work in *E. coli*, where Schelling and Conti found that outer membrane porins do not effect prey susceptibility^38^. OmpU is the most abundant outer membrane protein in *V. cholerae*^39^ and could have effects on early predator–prey interactions. *B. bacteriovorus* encodes 150 putative proteases^40^ and may use these enzymes to degrade outer membrane proteins and invade the prey cells. Thus it is possible that the less proteinaceous membrane of the ∆*ompU* strain may take longer to penetrate.

*B. bacteriovorus* has the potential to treat infections caused by many multidrug-resistant bacteria, including World Health Organization-designated priority pathogens and most of the ESKAPE pathogens^1^. The genetic mechanisms of prey resistance identified in this study may inform how *B. bacteriovorus* is applied to patients alone or used in combination therapies. For instance, we have shown that highly motile prey are more resistant to *B. bacteriovorus* predation. However, gut microbes are known to dampen flagella expression to avoid immune detection by the human host^41^, likely making them more susceptible to *B. bacteriovorus*. Additionally, this may bode well for *B. bacteriovorus*’ use in disrupting the non-motile bacteria in biofilms, as has been shown previously^42^.

In this study, we developed what we believe is the first flow cytometric assay to measure bacterial predator–prey interactions, as well as the software to analyze microscopic images for prey rounding by *B. bacteriovorus*. We hope that these two tools will be useful for research of both mutant prey and mutant predator phenotypes. Future studies may provide further insights into the mechanisms of resistance and sensitization for VC1087 and Opp permease genes.

## Methods

### Bacterial strains and growth conditions

A new *V. cholerae* strain was used in this study, HC1037 (GenBank accession: CP026647 and CP026648). This strain is of the O1 serogroup, Ogawa serotype, El Tor biotype, and was isolated from an adult patient at the Cholera Treatment Center in Jacmel, Haiti, on January 8, 2014. We deleted the endonuclease *ideA*^43^ in strain HC1037 to increase natural competence. Single-gene knockouts were constructed using the FRT/FLP recombinase method of deletion^18,19^. Complemented strains were created by cloning deleted genes and their native promoters into pMMB67EH^21^ and grown with ampicillin at 50 µg/ml to maintain the plasmid. Constitutive GFP-expressing *V. cholerae* was constructed via splicing-by-overlap extension (SOE) PCR and chitin-induced natural transformation. Briefly, three-piece SOE PCR was performed by amplifying an upstream region of homology with P172 and P61 (FR1), a downstream region of homology with P176 and P171 (FR3), and a middle product with P187 and P188 (FR2), which are designed to contain overlapping sequences with P61 and P176, respectively. The FR2 product includes the short half-life GFPASV while FR3 product contains a kanamycin-resistant marker. These SOE-PCR products were mixed together and serve as a template for PCR using P172 and P171. The final PCR product was added to *V. cholerae*, grown overnight on chitin to induce natural competence, and the next day transformants were selected for on antibiotic plates. Strains used in this study are shown in Supplementary Data 1. Primer sequences for gene deletions are listed in Supplementary Data 2. *V. cholerae* were grown overnight at 30 °C with shaking in LB Miller broth. *B. bacteriovorus* 109J and a red fluorescent strain expressing tdTomato on pMQ414 were used as predators in this study. Prey *E. coli* WM3064, a diaminopimelic acid (DAP) auxotroph, was grown overnight at 30 °C with 0.6 mM DAP and shaking. Predator stock lysates were prepared by coculturing prey cells with predators in 25 mM HEPES buffer amended with 3 mM MgCl_2_ and 2 mM CaCl_2_. For every 8 ml of HEPES buffer, 1 ml of *E. coli* overnight, resuspended in HEPES, and 1 ml of predator lysate were added. The coculture of prey and predator was incubated at 30 °C overnight with shaking for 24 h. Any remaining prey were removed by passing the lysate through a 0.45 µm filter. Gentamicin at 10 µg/ml was used to propagate the fluorescent *B. bacteriovorus* strain.

### Transposon library construction

Transposon libraries were created in *V. cholerae* and *E. coli* MG1655 using the temperature-sensitive mTn*10* delivery vector pDL1093 (Supplementary Figure 10) as described for pDL1098^16^. In brief, overnight cultures of *V. cholerae* or *E. coli* were grown in LB Miller broth, with shaking at 30 °C, with chloramphenicol at 10 µg/ml and kanamycin at 50 µg/ml to maintain the plasmid. The next day, 40 µl of the overnight culture was diluted into 400 ml of pre-warmed LB miller broth at 40 °C with kanamycin at 50 µg/ml. The temperature shift blocks plasmid replication and promotes transposition of m*Tn10* from the plasmid into the genome. The following day, stationary-phase cultures were collected and frozen as glycerol stocks for use in future Tn-seq experiments.

### Tn-seq screens during *B. bacteriovorus* predation

For the initial Tn-seq, *V. cholerae* and *E. coli* transposon libraries, previously frozen in glycerol, were outgrown overnight in LB broth at 30 °C with shaking. The next day, these prey bacteria were washed and resuspended in 25 mM HEPES buffer at 10^9^ CFU/ml. For the mini Tn-seq, each strain was grown individually overnight and pooled the following today. For each library outgrowth, 1 ml of prey was left uninfected, while another 1 ml tube was infected with fresh *B. bacteriovorus* at an MOI of 0.1 for *V. cholerae* and MOI 0.5 for *E. coli*. The infections proceeded at 30 °C with shaking for 3 h (*E. coli*) or 14 h (*V. cholerae*), sufficient for >99% prey killing. Following the co-incubation, the full 1 ml of surviving prey (≈5 × 10^6^ CFU) and 5 µl of the uninfected prey (≈5 × 10^6^ CFU) were outgrown separately overnight in 200 ml LB broth supplemented with kanamycin at 10 µg/ml at 30 °C with shaking. This outgrowth was necessary to recover enough cells for DNA isolation from both conditions. Genomic DNA was isolated using the DNeasy Blood and Tissue Kit (Qiagen).

The Tn-seq DNA was prepared for sequencing by a new method, Nextera Tn-seq, employing Nextera tagmentation (Illumina). In this method, the DNA was first tagmented using Tagment DNA enzyme 1 from the Nextera DNA Library Preparation Kit (FC-121-1030, Illumina). Next, nested PCR was carried out, first amplifying the region between the mTn*10* transposon (or FRT scar for the mini Tn-seq) and one of the Nextera transposomes. The second PCR added specific indices and Illumina end sequences needed for sequencing on the Illumina platform. The final PCR products were then pooled and purified using the QIAquick PCR Purification Kit (Qiagen). Primers for amplifying and indexing the samples are listed in Supplementary Data 2.

### Sequencing and fitness calculation

Pooled and indexed DNA samples were sequenced on the Illumina HiSeq 2500 using an mTn*10* or FRT scar-specific sequencing primer. The sequence reads of transposon or FRT junctions were analyzed using the Tufts University Core Facility Galaxy server. Reads were mapped to *V. cholerae* HC1037 (accession: CP026647 and CP026648) using Bowtie. The default Bowtie parameters were used, and the Tufts University Core Facility Galaxy server custom scripts “hopcount” and “aggregate hop table” were run on the mapped reads. Hopcount tallies the number of sequencing reads at every insertion site, which was used to determine the complexity of the input library, as well as the severity of the genetic selection. Aggregate hop table totals the number of sequencing reads in each gene. Included in the aggregate hop table output is a standardized frequency of transposon insertions for every gene (standardized read frequency), corrected for the gene size and calculated by the equation: standardized read frequency = [(number of reads in gene *X*/total number of reads in Illumina library)/(size of gene *X*/total genome size)].

### Competition assays

Following overnight growth, WT, deletion, or complemented *V. cholerae* strains were washed twice and resuspended at 10^9^ CFU/ml in HEPES buffer. Each strain was then mixed 1:1 with an isogenic ∆*lacZ* strain and split into two tubes. One tube was left uninfected, while the other was infected with *B. bacteriovorus* at MOI 0.1. Following 14 h of infection, the bacteria were serially diluted and plated for CFU/ml on LB with 80 µg/ml X-gal agar plates. For the increased viscosity competitions, Ficoll 400 (Sigma F8016) or 14 kD methylcellulose (Sigma M7140) were added at 10% and 1% final weight/volume, respectively. The competitive index was calculated as the ratio of the infected mutant and control strains normalized to the uninfected ratio of each strain.

### Flow cytometry

Overnight cultures of GFP-expressing *V. cholerae* were resuspended in HEPES to 10^8^ CFU/ml. Fresh cultures of tdTomato-expressing *B. bacteriovorus* were added to the *V. cholerae* at an MOI of 1. At 30 or 60 min postinfection, the bacteria were fixed in 1% formaldehyde for 10 min and diluted to 10^7^ CFU/ml in PBS. Three biological replicates were carried out for each sample. Attachment/colocalization was measured for the fluorescent *B. bacteriovorus* and *V. cholerae* using an S3e Cell Sorter (Bio-Rad). We used the FL1 and FL3 filters to detect GFP and tdTomato fluorescence, respectively. To confirm that our gating strategy correctly identified green-only (*V. cholerae*) and double-positive (*V. cholerae*+*B. bacteriovorus*) events, we sorted the populations into separate tubes and observed the cells by fluorescence microscopy (Supplementary Figure 4).

### Fluorescence microscopy

For the prey rounding experiment, overnight cultures of GFP-expressing *V. cholerae* were resuspended in HEPES to 10^8^ CFU/ml and infected with WT non-fluorescent *B. bacteriovorus* at MOI 1. Following 1 h of infection, the bacteria were fixed in 1% formaldehyde for 10 min. The bacteria were adhered to poly-L-lysine-coated slides using a Cytospin 3 (Shandon) at 450 × *g* for 1 min. Slides were imaged using a Nikon Eclipse 80i fluorescence microscope. Three images were taken per slide at ×40 magnification, each with approximately 500 bacteria present. In all, 3300–5000 bacteria were analyzed for each condition across the three biological replicates. For the predator invasion experiments, overnight cultures of GFP-expressing *V. cholerae* were resuspended in HEPES or HEPES with 10% ficoll to 10^8^ CFU/ml and infected with red fluorescent *B. bacteriovorus* at MOI 0.1. The cells were fixed and imaged as described above at 30 min and 1 h postinfection.

### Image analysis

For the prey rounding experiment, images were loaded into MATLAB and converted to double-precision grayscale arrays. These were converted to binary images, which were dilated and then eroded to smooth cell edges and remove background artifacts. Objects touching the edges of the field as well as objects <20 or >300 pixels were removed to exclude partial cells or aggregated cells from analysis. Eccentricity of the remaining cells was calculated using the MATLAB function regionprops. For the predator invasion experiment, images were scored, double blind, for invasion of *B. bacteriovorus* into *V. cholerae* or external attachment. We scored 200 *B. bacteriovorus* cells for each of three biological replicates.

### Viscosity measurements

We measured the kinematic viscosity of HEPES buffer alone, HEPES buffer with 10% ficoll, and HEPES buffer with 1% methylcellulose using Cannon–Fenske viscometers (Fungilab). All measurements were conducted with viscometers submerged in a water bath at 30 °C. The kinematic viscosity was converted to dynamic viscosity by multiplying kinematic viscosity by the liquid density.

### Calculations for drag and collision frequency

We estimated the frequency of collisions for *B. bacteriovorus* and WT or ∆*motY V. cholerae* strains using Eq. 1, which assumes that each species moves isotropically at a fixed velocity^33^:

1 $${\mathrm{Rate}}\,{\mathrm{of}}\,{\mathrm{predator\operatorname{-}prey}}\,{\mathrm{collisions}}\,{\mathrm{per}}\,{\mathrm{unit}}\,{\mathrm{volume}} \\ = n_{{\mathrm{predator}}}n_{{\mathrm{prey}}}\sigma \sqrt {\left( {v_{{\mathrm{predator}}}^2 + v_{{\mathrm{prey}}}^2} \right)}$$

Here *n*_predator_ and *n*_prey_ are the number of each bacteria per unit volume, and *σ* is the cross-sectional area for a collision to occur. For *B. bacteriovorus* we used a velocity (*v*) of 160 µm/s^4^, for WT *V. cholerae* we used 80 µm/s (Supplementary Figure 7), and for ∆*motY* we used 1 µm/s^26^. Assuming fixed values of *n*_predator_, *n*_prey_, and *σ*, we would expect 10% fewer collisions with *B. bacteriovorus* for the non-motile ∆*motY* strain compared to WT *V. cholerae* as a direct result of the lower average relative velocity. To calculate the drag force exerted on prey-attached *B. bacteriovorus*, we used Eq. 2:

2 $$F_{{\mathrm{drag}}} = 3{\mathrm{\pi \eta }}v(d_{\mathrm{n}})(1/3 + (2/3)d_{\mathrm{s}}/d_{\mathrm{n}})$$

Here *η* represents the medium’s dynamic viscosity, *v* is velocity, and *d*_n_ and *d*_s_ represent the equivalent spherical diameters of, respectively, the object’s cross-sectional area along the direction of motion and the object’s surface area. At 30 °C, HEPES medium has a viscosity of 0.77 cP, 10% ficoll has a viscosity of 4.03 cP, and 1% methylcellulose has a viscosity of 3.06 cP. For *B. bacteriovorus*, we estimate *d*_n_ to be 0.494 µm, and *d*_s_ to be 0.3 µm assuming *B. bacteriovorus* is reasonably approximated as a prolate spheroid with an equatorial radius of 0.15 µm and a polar radius of 0.5 µm. We used *V. cholerae* velocities of 80 µm/s (unattached speed) and 29.4 µm/s (observed speed with *B. bacteriovorus* attached).

### Statistical analysis

All statistical analysis was done on GraphPad Prism by ordinary one-way analysis of variance with Dunnett’s multiple comparisons test for significance. Data presented represent the mean with error bars signifying the standard error of the mean.

### Code availability

The MATLAB code to analyze cell rounding is available upon request.

## Electronic supplementary material

Supplementary Information

Description of Additional Supplementary Files

Supplementary Movie 1

Supplementary Movie 2

Supplementary Movie 3

Supplementary Movie 4

Supplementary Data 1

Supplementary Data 2

Supplementary Data 3

Supplementary Data 4

## Data Availability

The raw data that support the findings of this study are available from the corresponding author upon request.
