# Supplementary material for: Vibrio cholerae motility exerts drag force to impede attack by the bacterial predator Bdellovibrio bacteriovorus
Source: Nat Commun. 2018 Nov 12;9:4757. doi: 10.1038/s41467-018-07245-3 (PMC6232129; doi:10.1038/s41467-018-07245-3)
Supplement: Supplementary file 1 — Supplementary Information [file 41467_2018_7245_MOESM1_ESM.pdf]

Supplementary Information

Vibrio cholerae motility exerts drag force to impede attack by the bacterial predator *Bdellovibrio bacteriovorus*

Duncan *et al.*

Supplementary Figure 1a

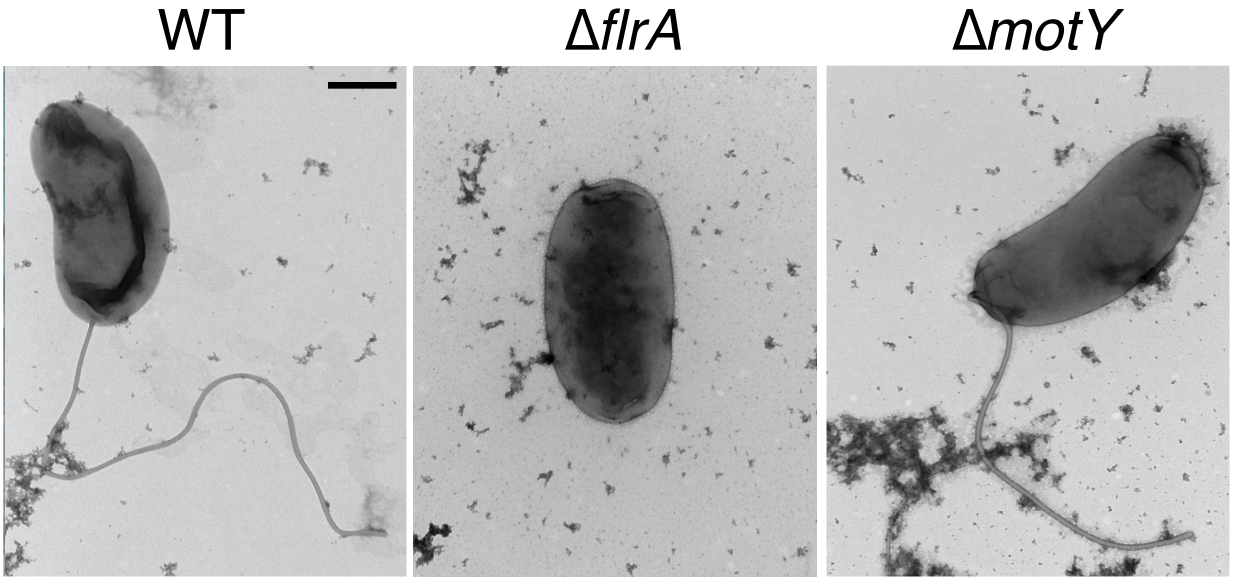

Supplementary Figure 1b

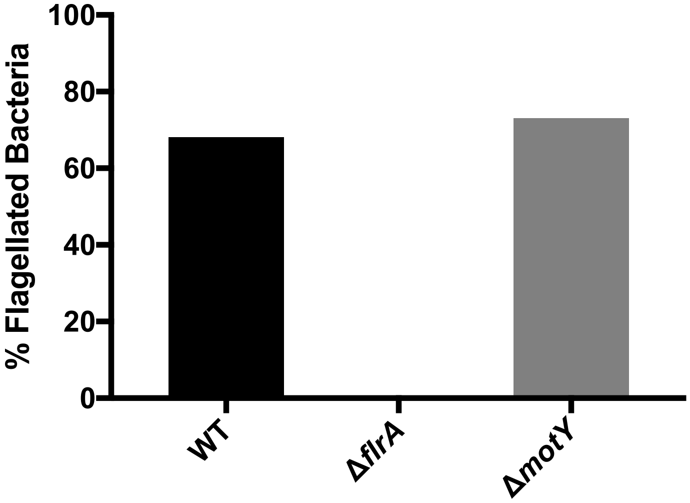

Supplementary Figure 1. The non-motile ΔflrA strain lacks flagella. **a**, Representative transmission electron microscopy images show the Δ*flrA* mutant lacks flagella, while Δ*motY* is also non-motile but retains its flagella. The scale bar indicates 500nm. **b**, The percent of flagellated bacteria for each strain is shown. Only one out of 113 Δ*flrA* bacteria possessed a flagellum.

Supplementary Figure 2

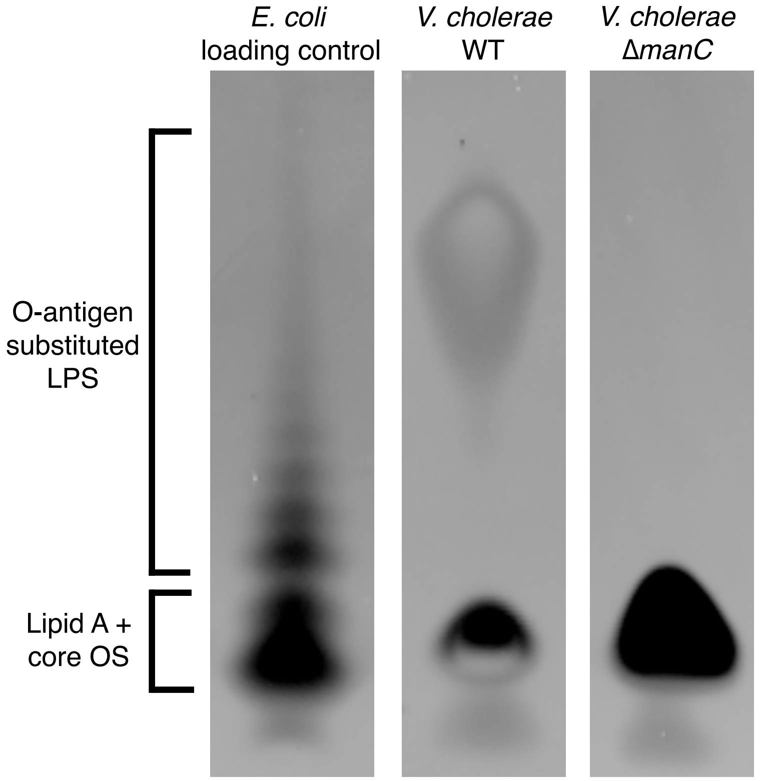

Supplementary Figure 2. The Δ*manC* strain does not produce O-antigen. Silver stain of an SDS-page gel following phenol extraction of LPS. Consistent with previous studies, the Δ*manC* mutant produces no observable O-antigen when compared to WT *V. cholerae*.

Supplementary Figure 3

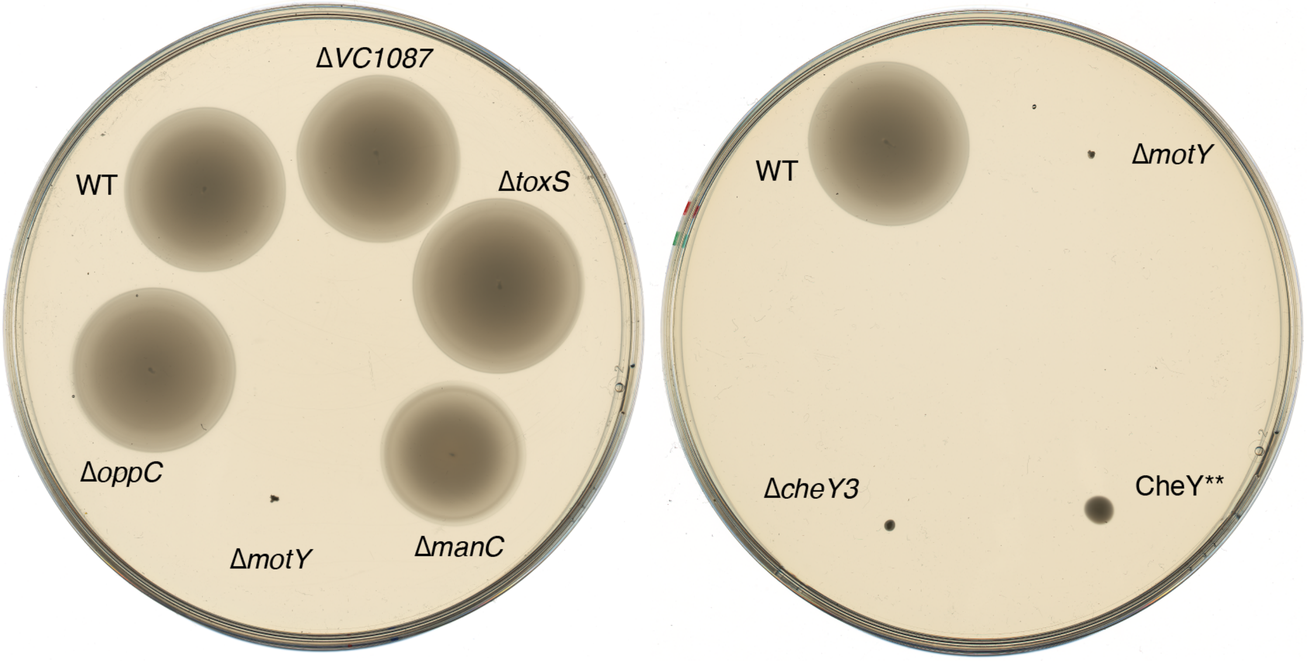

Supplementary Figure 3. Soft agar motility assay. Each mutant strain shows a similar swimming diameter compared to WT *V. cholerae* apart from the non-motile Δ*motY* strain and the chemotaxis mutant Δ*cheY3* and CheY** strains.

Supplementary Figure 4.

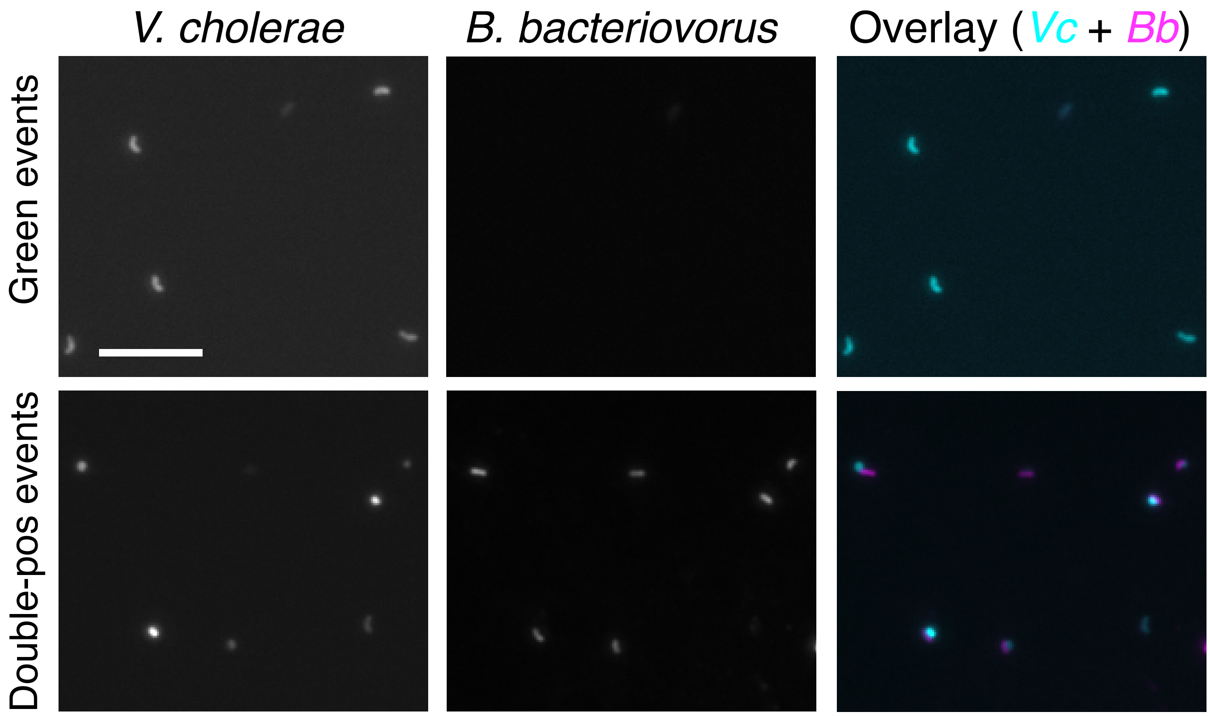

Supplementary Figure 4. Sorting and fluorescence microscopy to confirm flow cytometry distinguishes green-only and double-positive events. We sorted the two relevant populations into separate tubes and observed the cells by fluorescence microscopy. The gating strategy correctly identified green-only (*V. cholerae*) and double-positive (*V.* cholerae + *B. bacteriovorus*) events. In the overlay panels, *V. cholerae* are cyan and *B. bacteriovorus* are magenta.

Supplementary Figure 5

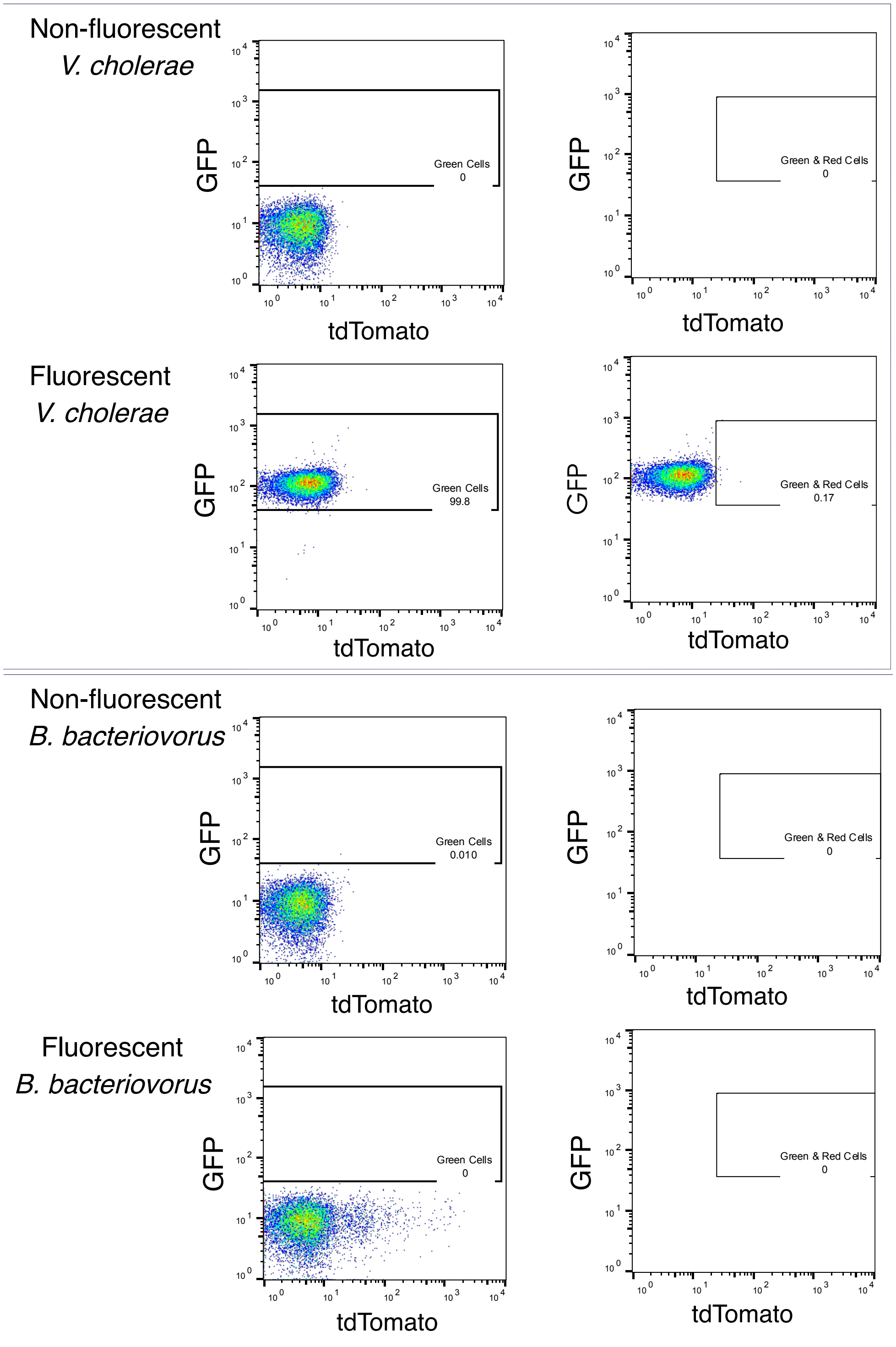

Supplementary Figure 5 Gating strategy for flow cytometry analysis of *B. bacteriovorus* interaction with *V. cholerae*. 10,000 events were collected for each sample. The gating strategy was designed to limit false-positive interactions.

Supplementary Figure 6

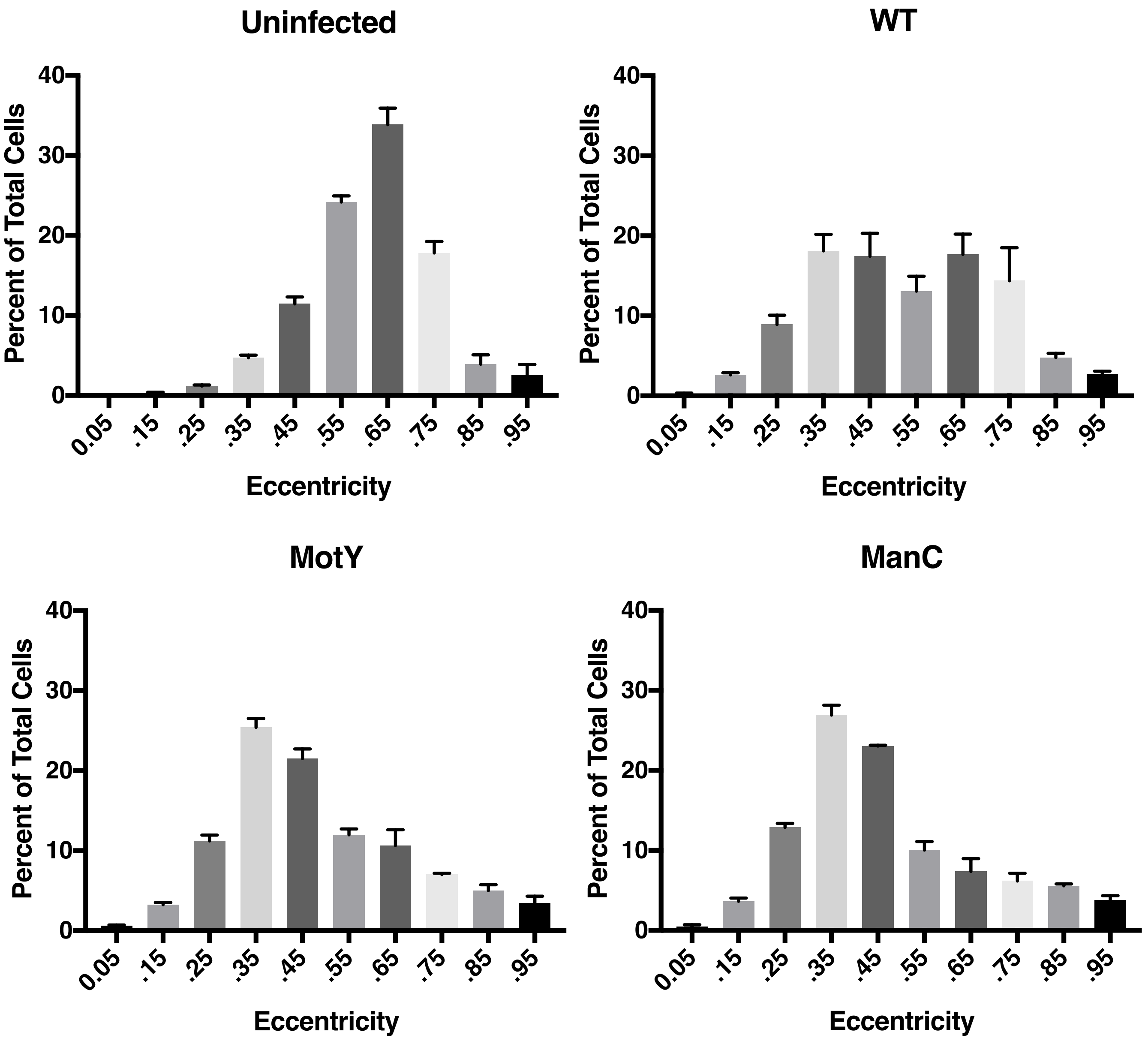

Supplementary Figure 6. Histograms of eccentricity values used in Fig 4b. Eccentricity values closer to zero indicate more rounded cells. Each bar represents cell values within 0.05 of the marked value. The average is shown for three biological replicates. Error bars represent standard error of the mean.

Supplementary Figure 7

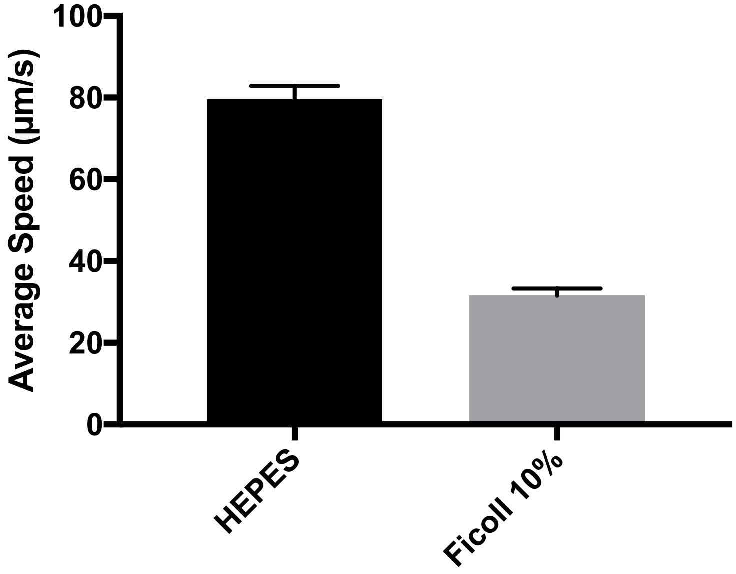

Supplementary Figure 7. 10% ficoll decreases *V. cholerae* swimming speed 2.5-fold. A 10% ficoll solution in HEPES reduces *V. cholerae* swimming speed from 80*µ*m/s to 32*µ*m/s. This 2.5-fold decrease in speed is less than the 6.5-fold increase in viscosity from adding ficoll. Individual bacteria were manually tracked using time-lapse microscopy. Error bars represent standard error of the mean. Each bar represents the average of three biological replicates.

Supplementary Figure 8a

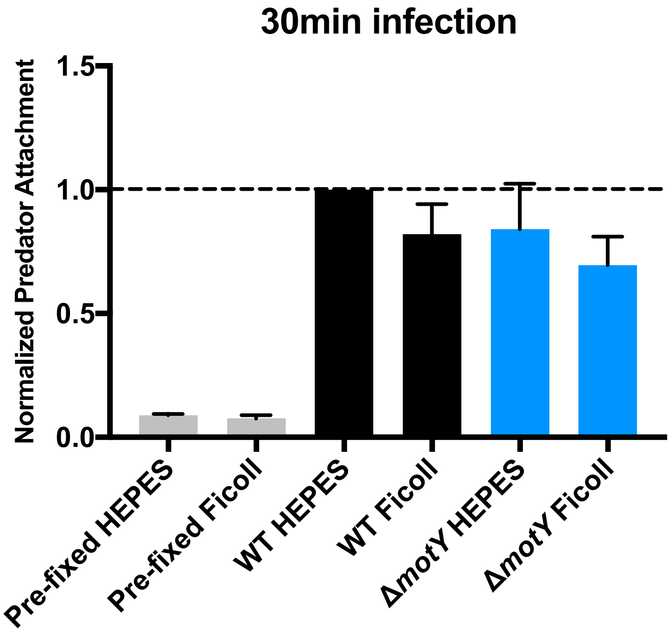

Supplementary Figure 8b

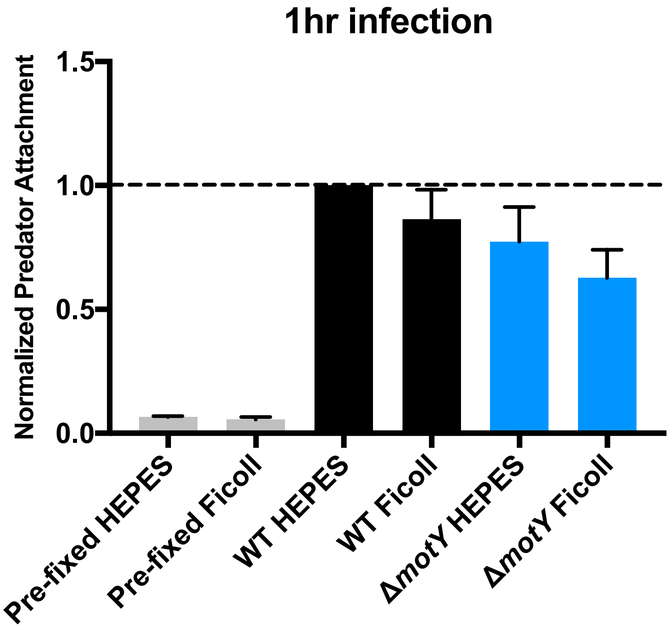

Supplementary Figure 8. Increased viscosity does not significantly reduce predator-prey attachment. Attachment was measured by flow cytometry at 30 minutes (**a**) or one hour (**b**) post infection in HEPES or 10% ficoll media. While both WT and Δ*motY V. cholerae* showed a slight decrease in attachment, only the motile WT strain demonstrates improved survival in the high viscosity medium. Each bar shows the average of three biological replicates. Error bars represent standard error of the mean.

Supplementary Figure 9a

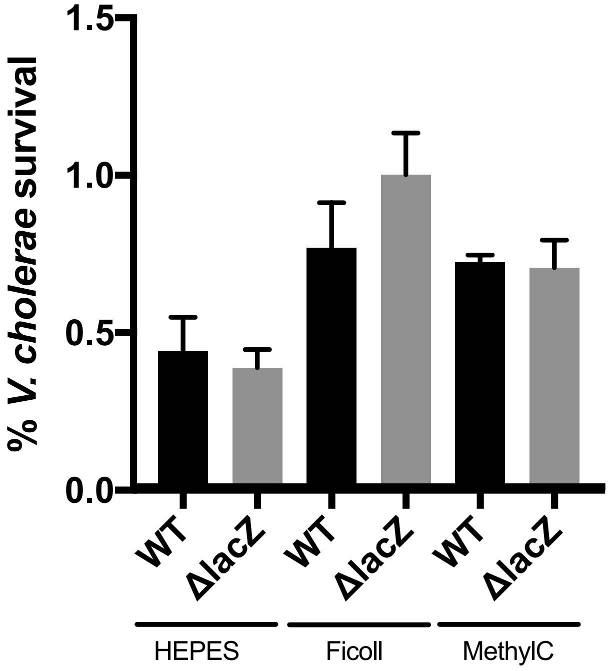

Supplementary Figure 9b

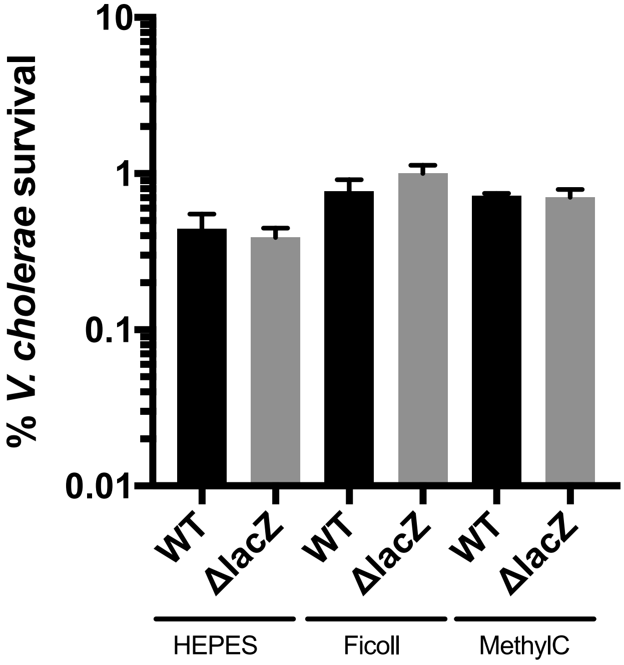

Supplementary Figure 9. Increased viscosity does not alter the competitive index between WT *V. cholerae* and an isogenic ΔlacZ strain. a-b, Survival percentages are shown for the WT vs. Δ*lacZ* controls from Fig. 4a and 4b. The survival rates are shown on a linear scale (a) and log scale (b) to allow comparison to the results in Figure 4a-b. Each bar shows the average of three biological replicates. Error bars represent standard error of the mean.

Supplementary Figure 10

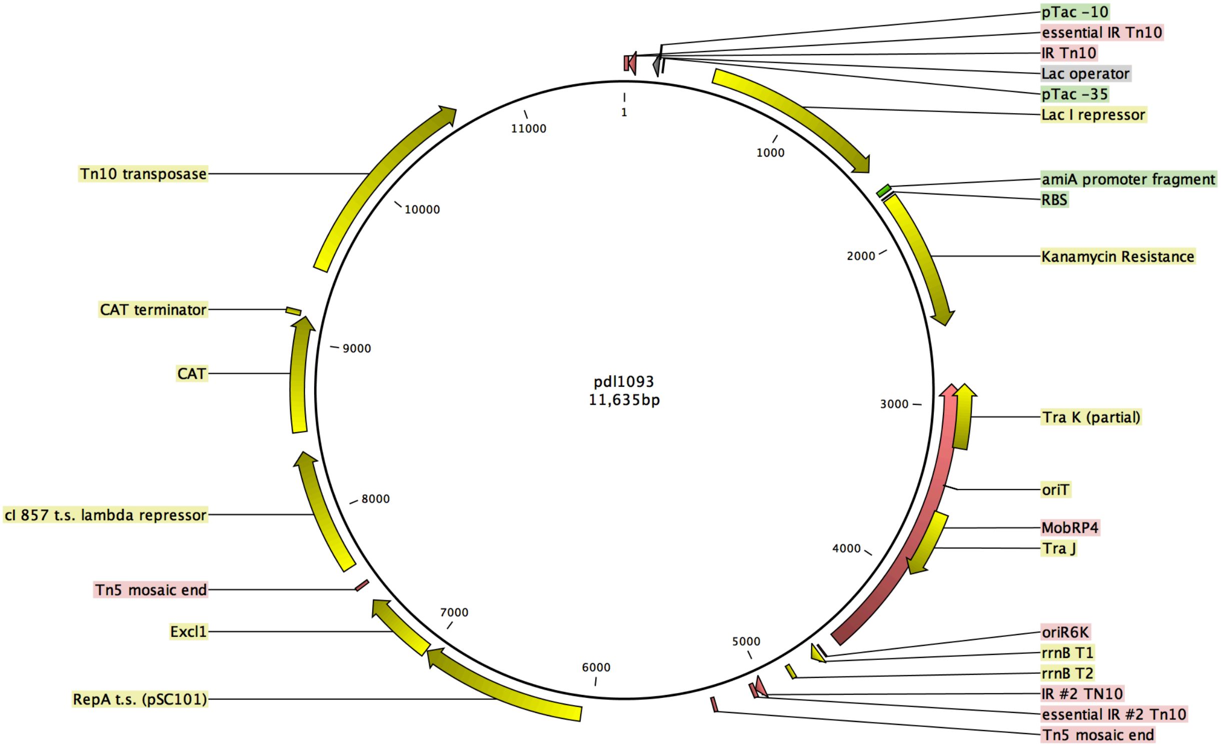

Supplementary Figure 10. pDL1093, a temperature-sensitive mTn*10* delivery vector.

Sequence:

CTGATGAATCCCCTAATGATTTTGGTAAAAATCATTAAGTTAAGGTGGATACACATC
TTGTCATATGTCGAGTGCCCACACAGATTGTCTGATAAATTGTTAAAGAGCAGTGCC
GCTTCGCTTTTTCTCAGCGGCGCCCCGAAACCATTTGATCCTGTTTCCTGTGTGAAA
TTGTTATCCGCTCACAATTCCACACATTATACGAGCCGATGATTAATTGTCAACAGC
TCATTTCAGAATATTTGCCAGAACCGTTATGATGTCGGCGCAAAAAACATTATCCAG
AACGGGAGTGCGCCTTGAGCGACACGAATTATGCAGTGATTTACGACCTGCACAG
CCATACCACAGCTTCCGATGGCTGCCTGACGCCAGAAGCATTGGTGCACCGTGCA
GTCGATGATAAGCTGTCAAACATGAGAATTCCCGGGAGAGCTCGGTACCCGACAC
CATCGAATGGTGCAAAACCTTTCGCGGTATGGCATGATAGCGCCCGGAAGAGAGT
CAATTCAGGGTGGTGAATGTGAAACCAGTAACGTTATACGATGTCGCAGAGTATGC
CGGTGTCTCTTATCAGACCGTTTCCCGCGTGGTGAACCAGGCCAGCCACGTTTCTG
CGAAAACGCGGGAAAAAGTGGAAGCGGCGATGGCGGAGCTGAATTACATTCCCAA
CCGCGTGGCACAACAACTGGCGGGCAAACAGTCGTTGCTGATTGGCGTTGCCACC
TCCAGTCTGGCCCTGCACGCGCCGTCGCAAATTGTCGCGGCGATTAAATCTCGCG
CCGATCAACTGGGTGCCAGCGTGGTGGTGTCGATGGTAGAACGAAGCGGCGTCGA
AGCCTGTAAAGCGGCGGTGCACAATCTTCTCGCGCAACGCGTCAGTGGGCTGATC
ATTAACTATCCGCTGGATGACCAGGATGCCATTGCTGTGGAAGCTGCCTGCACTAA

TGTTCCGGCGTTATTTCTTGATGTCTCTGACCAGACACCCATCAACAGTATTATTTT
CTCCCATGAAGACGGTACGCGACTGGGCGTGGAGCATCTGGTCGCATTGGGTCAC
CAGCAAATCGCGCTGTTAGCGGGCCCATTAAGTTCTGTCTCGGCGCGTCTGCGTCT
GGCTGGCTGGCATAAATATCTCACTCGCAATCAAATTCAGCCGATAGCGGAACGGG
AAGGCGACTGGAGTGCCATGTCCGGTTTTCAACAAACCATGCAAATGCTGAATGAG
GGCATCGTTCCCACTGCGATGCTGGTTGCCAACGATCAGATGGCGCTGGGCGCAA
TGCGCGCCATTACCGAGTCCGGGCTGCGCGTTGGTGCGGATATCTCGGTAGTGGG
ATACGACGATACCGAAGACAGCTCATGTTATATCCCGCCGTTAACCACCATCAAAC
AGGATTTTCGCCTGCTGGGGCAAACCAGCGTGGACCGCTTGCTGCAACTCTCTCA
GGGCCAGGCGGTGAAGGGCAATCAGCTGTTGCCCGTCTCACTGGTGAAAGAAAAA
CCACCCTGGCGCCCAATACGCAAACCGCCTCTCCCCGCGCGTTGGCCGATTCATT
AATGCAGCTGGCACGACAGGTTTCCCGACTGGAAAGCGGGCAGTGAGCGCAACGC
AATTAATGTAAGTTAGCTCACTCATTAGGCACCCCAGGCTTTACACTTTATGCTTCC
GACCTGCAGGTTTGATTTTTAATGGATAATGTGATATAATCTTTAAATACTGTAGAAA
AGAGGAAGGAAATAATAAATGGCTAAAATGAGAATATCACCGGAATTGAAAAAACT
GATCGAAAAATACCGCTGCGTAAAAGATACGGAAGGAATGTCTCCTGCTAAGGTAT
ATAAGCTGGTGGGAGAAAATGAAAACCTATATTTAAAAATGACGGACAGCCGGTAT
AAAGGGACCACCTATGATGTGGAACGGGAAAAGGACATGATGCTATGGCTGGAAG
GAAAGCTGCCTGTTCCAAAGGTCCTGCACTTTGAACGGCATGATGGCTGGAGCAAT
CTGCTCATGAGTGAGGCCGATGGCGTCCTTTGCTCGGAAGAGTATGAAGATGAACA
AAGCCCTGAAAAGATTATCGAGCTGTATGCGGAGTGCATCAGGCTCTTTCACTCCA
TCGACATATCGGATTGTCCCTATACGAATAGCTTAGACAGCCGCTTAGCCGAATTG
GATTACTTACTGAATAACGATCTGGCCGATGTGGATTGCGAAAACTGGGAAGAAGA
CACTCCATTTAAAGATCCGCGCGAGCTGTATGATTTTTTAAAGACGGAAAAGCCCG
AAGAGGAACTTGTCTTTTCCCACGGCGACCTGGGAGACAGCAACATCTTTGTGAAA
GATGGCAAAGTAAGTGGCTTTATTGATCTTGGGAGAAGCGGCAGGGCGGACAAGT
GGTATGACATTGCCTTCTGCGTCCGGTCGATCAGGGAGGATATCGGGGAAGAACA
GTATGTCGAGCTATTTTTTGACTTACTGGGGATCAAGCCTGATTGGGAGAAAATAAA
ATATTATATTTTACTGGATGAATTGTTTTAGGACGGGCTTGTCTGCTCCCGGCATCC
GCTTACAGACAAGCTGTGACCGTCTCCGGGAGCTGCATGTGTCAGAGGTTTTCACC
GTCATCACCGAAACGCGCGAGGCAGCAAGGAGATGGCGCCCAACAGTCCCCCGG
CCACGGGGCCTGCCACCATACCCACGCCGAAACAAGCGCTCATGAGCCCGAAGTG
GCGAGCCCGATCTTCCCCATCGGTGATGTCGGCGATATAGGCGCCAGCAACCGCA
CCTGTGGCGCCGGTGATGCCGGCCACGATGCGTCCGGCGTAGAGGATCCTTTTTG
TCCGGTGTTGGGTTGAAGGTGAAGCCGGTCGGGGCCGCAGCGGGGGCCGGCTTT
TCAGCCTTGCCCCCCTGCTTCGGCCGCCGTGGCTCCGGCGTCTTGGGTGCCGGC
GCGGGTTCCGCAGCCTTGGCCTGCGGTGCGGGCACATCGGCGGGCTTGGCCTTG
ATGTGCCGCCTGGCGTGCGAGCGGAACGTCTCGTAGGAGAACTTGACCTTCCCCG
TTTCCCGCATGTGCTCCCAAATGGTGACGAGCGCATAGCCGGACGCTAACGCCGC
CTCGACATCCGCCCTCACCGCCAGGAACGCAACCGCAGCCTCATCACGCCGGCGC
TTCTTGGCCGCGCGGGATTCAACCCACTCGGCCAGCTCGTCGGTGTAGCTCTTTG
GCATCGTCTCTCGCCTGTCCCCTCAGTTCAGTAATTTCCTGCATTTGCCTGTTTCCA
GTCGGTAGATATTCCACAAAACAGCAGGGAAGCAGCGCTTTTCCGCTGCATAACCC
TGCTTCGGGGTCATTATAGCGATTTTTTCGGTATATCCATCCTTTTTCGCACGATAT
ACAGGATTTTGCCAAAGGGTTCGTGTAGACTTTCCTTGGTGTATCCAACGGCGTCA

GCCGGGCAGGATAGGTGAAGTAGGCCCACCCGCGAGCGGGTGTTCCTTCTTCACT
GTCCCTTATTCGCACCTGGCGGTGCTCAACGGGAATCCTGCTCTGCGAGGCTGGC
CGGCTACCGCCGGCGTAACAGATGAGGGCAAGCGGATGGCTGATGAAACCAAGC
CAACCAGGAAGGGCAGCCCACCTATCAAGGTGTACTGCCTTCCAGACGAACGAAG
AGCGATTGAGGAAAAGGCGGCGGCGGCCGGCATGAGCCTGTCGGCCTACCTGCT
GGCCGTCGGCCAGGGCTACAAAATCACGGGCGTCGTGGACTATGAGCACGTCCGC
GAGCTGGCCCGCATCAATGGCGACCTGGGCCGCCTGGGCGGCCTGCTGAAACTC
TGGCTCACCGACGACCCGCGCACGGCGCGGTTCGGTGATGCCACGATCCTCGCC
CTGCTGGCGAAGATCGAAGAGAAGCAGGACGAGCTTGGCAAGGTCATGATGGGCG
TGGTCCGCCCGAGGGCAGAGCCATGACTTTTTTAGCCGCTAAAACGGCCGGGGGG
TGCGCGTGATTGCCAAGCACGTCCCCATGCGCTCCATCAAGAAGAGCGACTTCGC
GGAGCTGGTGAAGTACATCACCGACGAGCAAGGCAAGACCGAGCGCCTGGGTCA
CGTGCGCGTCACGAACTGCGAGGCAAACACCCTGCCCGCTGTCATGGCCGAGGTG
ATGGCGACCCAGCACGGCAACACCCGTTCCGAGGCCGACAAGACCTATCACCTGC
TGGTTAGCTTCCGCGCGGGAGAGAAGCCCGACGCGGAGACGTTGCGCGCGATTG
AGGACCGCATCTGCGCTGGGCTTGGCTTCGCCGAGCATCAGCGCGTCAGTGCCGT
GCATCACGACACCGACAACCTGCACATCCATATCGCCATCAACAAGATTCACCCGA
CCCGAAACACCATCCATGAGCCGTATCGGGCCTACCGCGCCCTCGCTGACCTCTG
CGCGACGCTCGAACGGGACTACGGGCTTGAGCGTGACAATCACGAAACGCGGCA
GCGCGTTTCCGAGAACCGCGCGAACGACATGGAGCGGCACGCGGGCGTGGAAAG
CCTGGTCGGCTGGATCCGGCCACGATGCGTCCGGCGTAGAGGATCTGAAGATCAG
CAGTTCAACCTGTTGATAGTACGTCGCCGGCGGCATCAAATAAAACGAAAGGCTCA
GTCGAAAGACTGGGCCTTTCGTTTTATCTGTTGTTTGTCGGTGAACGCTCTCCTGA
GTAGGACAAATCCGCCGGGAGCGGATTTGAACGTTGCGAAGCAACGGCCCGGAG
GGTGGCGGGCAGGACGCCCGCCATAAACTGCCAGGCATCAAATTAAGCAGAAGGC
CATCCTGACGGATGGCCTTTTTGCGTTTCTACAAACTCTTTTGTTTATTTTTGTCGAC
TGTACAGCGGCCGCGGCCTAGGCGGCCACGCGTATTCAGGCTGACCCTGCGCGC
TGCGCAGGGCTTTATTGATTCCATTTTTACACTGATGAATGTTCCGTTGCGCTGCCC
GGATTACAGCCGGATCCGGGATCATATGACAAGATGTGTATCCACCTTAACTTAAT
GATTTTTACCAAAATCATTAGGGGATTCATCAGTGCTCAGGGTCAACGAGAATTAAC
ATTCCGTCAGGAAAGCTTATGATGATGATGTGCTTAAAAACTTACTCAATGGCTGGT
TTATGCAGATATCCATCACACTGGCGGCCGCTCGAGCATGCGTCGACTCTAGAGGA
TCCGAGCTACTCCGTTACAAAGCGAGGCTGGGTATTTCCCGGCCTTTCTGTTATCC
GAAATCCACTGAAAGCACAGCGGAGATGTGTATAAGAGACAGCTGGACAGTAAGA
CGGGTAAGCCTGTTGATGATACCGCTGCCTTACTGGGTGCATTAGCCAGTCTGAAT
GACCTGTCACGGGATAATCCGAAGTGGTCAGACTGGAAAATCAGAGGGCAGGAAC
TGCTGAACAGCAAAAAGTCAGATAGCACCACATAGCAGACCCGCCATAAAACGCCC
TGAGAAGCCCGTGACGGGCTTTTCTTGTATTATGGGTAGTTTCCTTGCATGAATCCA
TAAAAGGCGCCTGTAGTGCCATTTACCCCCATTCACTGCCAGAGCCGTGAGCGCA
GCGAACTGAATGTCACGAAAAAGACAGCGACTCAGGTGCCTGATGGTCGGAGACA
AAAGGAATATTCAGCGATTTGCCCGAGCTTGCGAGGGTGCTACTTAAGCCTTTAGG
GTTTTAAGGTCTGTTTTGTAGAGGAGCAAACAGCGTTTGCGACATCCTTTTGTAATA
CTGCGGAACTGACTAAAGTAGTGAGTTATACACAGGGCTGGGATCTATTCTTTTTAT
CTTTTTTTATTCTTTCTTTATTCTATAAATTATAACCACTTGAATATAAACAAAAAAAA
CACACAAAGGTCTAGCGGAATTTACAGAGGGTCTAGCAGAATTTACAAGTTTTCCA

GCAAAGGTCTAGCAGAATTTACAGATACCCACAACTCAAAGGAAAAGGACTAGTAA
TTATCATTGACTAGCCCATCTCAATTGGTATAGTGATTAAAATCACCTAGACCAATT
GAGATGTATGTCTGAATTAGTTGTTTTCAAAGCAAATGAACTAGCGATTAGTCGCTA
TGACTTAACGGAGCATGAAACCAAGCTAATTTTATGCTGTGTGGCACTACTCAACCC
CACGATTGAAAACCCTACAAGGAAAGAACGGACGGTATCGTTCACTTATAACCAAT
ACGCTCAGATGATGAACATCAGTAGGGAAAATGCTTATGGTGTATTAGCTAAAGCA
ACCAGAGAGCTGATGACGAGAACTGTGGAAATCAGGAATCCTTTGGTTAAAGGCTT
TGAGATTTTCCAGTGGACAAACTATGCCAAGTTCTCAAGCGAAAAATTAGAATTAGT
TTTTAGTGAAGAGATATTGCCTTATCTTTTCCAGTTAAAAAAATTCATAAAATATAAT
CTGGAACATGTTAAGTCTTTTGAAAACAAATACTCTATGAGGATTTATGAGTGGTTA
TTAAAAGAACTAACACAAAAGAAAACTCACAAGGCAAATATAGAGATTAGCCTTGAT
GAATTTAAGTTCATGTTAATGCTTGAAAATAACTACCATGAGTTTAAAAGGCTTAACC
AATGGGTTTTGAAACCAATAAGTAAAGATTTAAACACTTACAGCAATATGAAATTGG
TGGTTGATAAGCGAGGCCGCCCGACTGATACGTTGATTTTCCAAGTTGAACTAGAT
AGACAAATGGATCTCGTAACCGAACTTGAGAACAACCAGATAAAAATGAATGGTGA
CAAAATACCAACAACCATTACATCAGATTCCTACCTACATAACGGACTAAGAAAAAC
ACTACACGATGCTTTAACTGCAAAAATTCAGCTCACCAGTTTTGAGGCAAAATTTTT
GAGTGACATGCAAAGTAAGTATGATCTCAATGGTTCGTTCTCATGGCTCACGCAAA
AACAACGAACCACACTAGAGAACATACTGGCTAAATACGGAAGGATCTGAGGTTCT
TATGGCTCTTGTATCTATCAGTGAAGCATCAAGACTAACAAACAAAAGTAGAACAAC
TGTTCACCGTTACATATCAAAGGGAAAACTGTCCATATGCACAGATGAAAACGGTGT
AAAAAAGATAGATACATCAGAGCTTTTACGAGTTTTTGGTGCATTCAAAGCTGTTCA
CCATGAACAGATCGACAATGTAACAGATGAACAGCATGTAACACCTAATAGAACAG
GTGAAACCAGTAAAACAAAGCAACTAGAACATGAAATTGAACACCTGAGACAACTT
GTTACAGCTCAACAGTCACACATAGACAGCCTGAAACAGGCGATGCTGCTTATCGA
ATCAAAGCTGCCGACAACACGGGAGCCAGTGACGCCTCCCGTGGGGAAAAAATCA
TGGCAATTCTGGAAGAAATAGCGCTTTCAGCCGGCAAACCTGAAGCCGGATCTGC
GATTCTGATAACAAACTAGCAACACCAGAACAGCCCGTTTGCGGGCAGCAAAACCC
GTCAGCTGTCTCTTATACACATCTCGTTGTTCCATACAACCTCCTTAGTACATGCAA
CCATTATCACCGCCAGAGGTAAAATAGTCAACACGCACGGTGTTAGATATTTATCCC
TTGCGGTGATAGATTTAACGTATGAGCACAAAAAAGAAACCATTAACACAAGAGCA
GCTTGAGGACGCACGTCGCCTTAAAGCAATTTATGAAAAAAAGAAAAATGAACTTG
GCTTATCCCAGGAATCTGTCGCAGACAAGATGGGGATGGGGCAGTCAGGCGTTGG
TGCTTTATTTAATGGCATCAATGCATTAAATGCTTATAACGCCGCATTGCTTACAAAA
ATTCTCAAAGTTAGCGTTGAAGAATTTAGCCCTTCAATCGCCAGAGAAATCTACGAG
ATGTATGAAGCGGTTAGTATGCAGCCGTCACTTAGAAGTGAGTATGAGTACCCTGT
TTTTTCTCATGTTCAGGCAGGGATGTTCTCACCTAAGCTTAGAACCTTTACCAAAGG
TGATGCGGAGAGATGGGTAAGCACAACCAAAAAAGCCAGTGATTCTGCATTCTGGC
TTGAGGTTGAAGGTAATTCCATGACCGCACCAACAGGCTCCAAGCCAAGCTTTCCT
GACGGAATGTTAATTCTCGTTGACCCTGAGCAGGCTGTTGAGCCAGGTGATTTCTG
CATAGCCAGACTTGGGGGTGATGAGTTTACCTTCAAGAAACTGATCAGGGATAGCG
GTCAGGTGTTTTTACAACCACTAAACCCACAGTACCCAATGATCCCATGCAATGAGA
GTTGTTCCGTTGTGGGGAAAGTTATCGCTAGTCAGTGGCCTGAAGAGACGTTTGGC
TGATCGGCAAGGTGTTCTGGTCGGCGCATAGCTGATAACAATTGAGCAAGAATCTT
CATCGAATTAGGGGAATTTTCACTCCCCTCAGAACATAACATAGTAAATGGATTGAA

TTATGGAGAAAAAAATCACTGGATATACCACCGTTGATATATCCCAATGGCATCGTA
AAGAACATTTTGAGGCATTTCAGTCAGTTGCTCAATGTACCTATAACCAGACCGTTC
AGCTGGATATTACGGCCTTTTTAAAGACCGTAAAGAAAAATAAGCACAAGTTTTATC
CGGCCTTTATTCACATTCTTGCCCGCCTGATGAATGCTCATCCGGAATTCCGTATG
GCAATGAAAGACGGTGAGCTGGTGATATGGGATAGTGTTCACCCTTGTTACACCGT
TTTCCATGAGCAAACTGAAACGTTTTCATCGCTCTGGAGTGAATACCACGACGATTT
CCGGCAGTTTCTACACATATATTCGCAAGATGTGGCGTGTTACGGTGAAAACCTGG
CCTATTTCCCTAAAGGGTTTATTGAGAATATGTTTTTCGTCTCAGCCAATCCCTGGG
TGAGTTTCACCAGTTTTGATTTAAACGTGGCCAATATGGACAACTTCTTCGCCCCCG
TTTTCACCATGGGCAAATATTATACGCAAGGCGACAAGGTGCTGATGCCGCTGGCG
ATTCAGGTTCATCATGCCGTCTGTGATGGCTTCCATGTCGGCAGAATGCTTAATGA
ATTACAACAGTACTGCGATGAGTGGCAGGGCGGGGCGTAACCCGGCCTCAGCGCC
GGGTTTTCTTTGCCTCACGATCGCCCCCAAAACACATAACCAATTGTATTTATTGAA
AAATAAATAGATACAACTCACTAAACATAGCAATTCAGATCTCTCACCTACCAAACA
ATGCCCCCCTGCAAAAAATAAATTCATATAAAAAACATACAGATAACCATCTGCGGT
GATAAATTATCTCTGGCGGTGTTGACATAAATACCACTGGCGGTGATACTGAGCAC
ATCAGCGAGCTCAAAAATCAATAATCAGACAACAAGATGTGCGAACTCGATATTTTA
CACGACTCTCTTTACCAATTCTGCCCCGAATTACACTTAAAACGACTCAACAGCTTA
ACGTTGGCTTGCCACGCATTACTCAATTGTAAAACTCTCACTCTTACCGAACTTGGC
CGTAACCTGCCAACCAAAGCGAGAACAAAACATAACATCAAACGAATCGACCGATT
GTTAGGTAATCGTCACCTCCACAAAGAGCGACTCGCTGTATACCGTTGGCATGCTA
GCTTTATCTGTTCGGGCAATACGATGCCCATTGTACTTGTTGACTGGTCTGATATTC
GTGAGCAAAAACGACTTATGGTATTGCGAGCTTCAGTCGCACTACACGGTCGTTCT
GTTACTCTTTATGAGAAAGCGTTCCCGCTTTCAGAGCAATATTCAAAGAAAGCTCAT
GACCAATTTCTAGCCGACCTTGCGAGCATTCTACCGAGTAACACCACACCGCTCAT
TGTCAGTGATGCTGGCTTTAAAGTGCCATGGTATAAATCCGTTGAGAAGCTGGGTT
GGTACTGGTTAAGTCGAGTAAGAGGAAAAGTACAATATGCAGACCTAGGAGCGGAA
AACTGGAAACCTATCAGCAACTTACATGATATGTCATCTAGTCACTCAAAGACTTTA
GGCTATAAGAGGCTGACTAAAAGCAATCCAATCTCATGCCAAATTCTATTGTATAAA
TCTCGCTCTAAAGGCCGAAAAAATCAGCGCTCGACACGTACGCATTATCACCACCC
GTCACCTAAAATCTACTCAGCGTCGGCAAAGGAGCCATGGGTTCTAGCAACTAACT
TACCTGTTGAAATTCGGACACCCAAACAACTTGTTAATATCTATTCGAAGCGAATGC
AGATTGAAGAAACCTTCCGAGACTTGAAAAGTCCTGCCTACGGACTAGGCCTACGC
CATAGCCGAACGAGCAGCTCAGAGCGTTTTGATATCATGCTGCTAATCGCCCTGAT
GCTTCAACTAACATGTTGGCTTGCGGGCGTTCATGCTCAGAAACAAGGTTGGGACA
AGCACTTCCAGGCTAACACAGTCAGAAATCGAAACGTACTCTCAACAGTTCGCTTA
GGCATGGAAGTTTTGCGGCATTCTGGCTACACAATAACAAGGGAAGACTTACTCGT
GGCTGCAACCCTACTAGCTCAAAATTTATTCACACATGGTTACGCTTTGGGGAAATT
ATGAGGGGATCTCTCAGTGCATTGCCTCCAATTCCCATAATTTATTACGCCGATAAT
AACTTGGTGTAACCTTAAAAATGTACTTAAATCGACGTGTAAAAGATTGTTGGGAAT
CAAATTGATATTTTAATGCGATCTCAAGGATAGTTTTTTTCGTCAACCTCAACTCAAC
AGCCGCTTTCGTCAAACGACGAGCACGAATATAGCTAGCCAGTGTGACCCCTGTTA
CTTTTTGAACAGCCGCTGAAAATACCACTTGGTATAACCCGCTTTATTCGCCACATC
ATCAAGCAGTAAAGACTGATCTAAATTATGTTCAATCCATAATAGAACATCTTTGATA
ACCGTTGTATGAAACTGCTTATCATCATATCTTAATTGGATGTTATTAGCTTTATTTT

GATAGCGAGAATGCTGTTCAATATACATAAAATAACCTAAATGTTCTTAAGATTGTCA
CGACCACATCATCATGATACCATAAACATACTGACGGTATGTTATTTTAAATCTATCA
TGGAAAATAAAAATCATCAACAAGAAAATTTTAAGAGTACCTATCAATCACTGGTTAA
CTCAGCACGAATATTGTTTGTTGAAAAAGGCTATCAAGCTGTTTCAATAGATGAGAT
CGATCTCGGGAAAAGCGTTGGTGACCAAAGGTGCCTTTTATCATCACTTTAAAAATA
AAAAACAATTACTCAGTGCCTGTTATAAGCAGCAATTAATTATGATTGATGCCTACAT
CACAACAAAAACTGATTTAACAAATGGTTGGTCTGCCTTAGAAAGTATATTTGAACA
TTATCTTGATTATATTATTGATAATAATAAAAACCTTATCCCTATCCAAGAAGTGATG
CCTATCATTGGTTGGAATGAACTTGAAAAAATTAGCCTTGAATACATTACTGGTAAG
GTAAACGCCATTGTCAGCAAATTGATCCAAGAGAACCAACTTAAAGCTTTCCTGACG
GAATGTTAATTCTCGTTGACCCTGAGCA

Supplementary Methods

Transmission electron microscopy (TEM). TEM was performed as previously described[1] using overnight cultures of cells grown in LB broth, washed with PBS, spotted on formvar coated grids, strained with uranyl acetate (1%) and viewed with a JEM-1210 electron microscope. At least 10 individual bacteria were imaged for each of the *V. cholerae* strains.

Phenol extraction of LPS. LPS was extracted and analyzed as previously described[2]. Briefly, phenol extraction was performed using phase-lock gel light tubes (Eppendorf). Extracts were centrifuged at 75,000× g for 60 min, the pellet was washed with TM buffer and centrifuged as before. Purified LPS was separated on a 4–12% NuPage Bis-Tris gel (Invitrogen) and visualized by silver-staining (SilverQuest, Invitrogen). The concentration of *V. cholerae* LPS was determined by comparison to a standard curve of *E. coli* O26:B6 LPS (Sigma) using a Fujifilm FLA-900 scanner.

***V. cholerae* speed determination.** Bacterial speed was determined as previously described[3]. Briefly, overnight *V. cholerae* cultures were resuspended in HEPES buffer or HEPES buffer with 10% Ficoll 400. Samples were imaged using dark-field microscopy at 40x magnification using a Nikon Eclipse 80i fluorescence microscope. Time-lapse images were taken every 0.4 seconds. At least 40 individual bacteria were manually tracked in the movies using ImageJ, and these distances were compared to the number of frames observed to calculate *V. cholerae* speed in μm/ second.

Movies of *V. cholerae* dragging *B. bacteriovorus*. We infected *V. cholerae* at MOI 10 with *B. bacteriovorus* in HEPES buffer as done for previous experiments. After 20 minutes of infection, we diluted the samples 1:10 into HEPES buffer and observed the predator-prey interactions using a Revolve microscope (Echo) at 90x magnification. We used iMovie software (Apple) to slow the movies to 0.25x full speed and insert annotation.

Supplementary References

1 Kalivoda, E. J., Brothers, K. M., Stella, N. A., Schmitt, M. J. & Shanks, R. M. Bacterial cyclic AMP-phosphodiesterase activity coordinates biofilm formation. *PLoS One* **8**, e71267, doi:10.1371/journal.pone.0071267 (2013).

2 Seed, K. D. *et al.* Phase variable O antigen biosynthetic genes control expression of the major protective antigen and bacteriophage receptor in *Vibrio cholerae* O1. *PLoS Pathog* **8**, e1002917, doi:10.1371/journal.ppat.1002917 (2012).

3 Butler, S. M. & Camilli, A. Both chemotaxis and net motility greatly influence the infectivity of *Vibrio cholerae*. *Proc Natl Acad Sci U S A* **101**, 5018-5023, doi:10.1073/pnas.0308052101 (2004).
