# Supplementary material for: Vibrio cholerae motility exerts drag force to impede attack by the bacterial predator Bdellovibrio bacteriovorus
Source: Nat Commun. 2018 Nov 12;9:4757. doi: 10.1038/s41467-018-07245-3 (PMC6232129; doi:10.1038/s41467-018-07245-3)
Supplement: Supplementary file 2 — Description of Additional Supplementary Files [file 41467_2018_7245_MOESM2_ESM.docx]

**Description of Additional Supplementary Files**

**File Name**: Supplementary Movie 1.

**Description:** A wide view at 90x magnification of V. cholerae infected with B. bacteriovorus played at full speed.

**File Name**: Supplementary Movie 2.

**Description**: A zoomed-in portion of Movie 1, originally at 90x magnification, shown at 0.25x speed to improve visualization. The red arrow indicates a predator-prey interaction of interest.

**File Name**: Supplementary Movie 3.

**Description**: A zoomed-in portion of a different movie, originally at 90x magnification, shown at 0.25x speed to improve visualization. The red arrow indicates a predator-prey interaction of interest.

**File Name**: Supplementary Movie 4.

**Description**: A zoomed-in view of uninfected V. cholerae, originally at 90x magnification, at 0.25x speed, for comparison to Supplementary Movies 2 and 3.

**File Name:** Supplementary Data 1.

**Description:** The list of bacterial strains used in this study.

**File Name:** Supplementary Data 2.

**Description:** The list of primer sequences used in this study.

**File Name:** Supplementary Data 3.

**Description:** Vibrio cholerae Tn-seq result table. Relative fitness for transposon-insertion

mutants during predation is shown for three biological replicates.

**File Name:** Supplementary Data 4.

**Description:** Escherichia coli Tn-seq result table. Relative fitness for transposoninsertion

mutants during predation is shown for three biological replicates.

**File Name:** Featured Image

**Description**: Fluorescence microscopy image of predatory Bdellovibrio bacteriovorus

(magenta) infecting its prey, Vibrio cholerae (cyan). Many B. bacteriovorus are attached

to V. cholerae, or have caused V. cholerae to round into bdelloplasts.
